# Comprehensive single-cell transcriptomic reveals different destinies of melanocytes and dynamic changes of immune microenvironment in a psychological stress-induced leukoderma and leukotrichia mouse model

**DOI:** 10.1186/s10020-025-01236-z

**Published:** 2025-05-14

**Authors:** Xuechen Cao, Yongkai Yu, Hang Yao, Yujie Zheng, Jiawei Lu, Yifei Feng, Tongxin Pei, Ziyu Li, Ming Lu, Yan Lu

**Affiliations:** 1 Department of Dermatology, Jiangsu Province Hospital and Nanjing Medical University First Affiliated Hospital, Nanjing, Jiangsu 210029 China; 2 Department of Pharmacology, Jiangsu Key Laboratory of Neurodegeneration, Nanjing Medical University, Nanjing, Jiangsu 211166 China; 3 Engineering Research Centre of Intelligent Theranostics Technology and Instruments, Ministry of Education, Nanjing, Jiangsu 210029 China; 4 Department of Dermatology, Jiangsu Province Hospital and Nanjing Medical University First Affiliated Hospital, Jiangsu Province, 210000 China

**Keywords:** Chronic unpredictable mild stress, Single-cell sequencing, Melanocyte stem cells, CXCL16, CXCR6, Innate immunity

## Abstract

**Background:**

Vitiligo is an acquired skin depigmentation disorder often accompanied by leukoderma and leukotrichia. Half of vitiligo patients experience episodes of stress.

**Methods:**

We established a chronic unpredictable mild stimulation (CUMS) model in C57BL/6 J mice to simulate chronic mental stress-induced leukoderma and leukotrichia. Single-cell RNA sequencing was performed to determine the immune landscape and to characterize the relationship between immune-stromal cells. Immunohistochemistry was employed for validation.

**Results:**

We discovered a similar pro-inflammatory micro-environment composed of keratinocytes and fibroblasts similar to that in human vitiligo. Macrophages in CUMS mice expressed high levels of inflammatory factors and were inclined to an M1 pro-inflammatory phenotype. Two distinct clusters of melanocytes were also identified: Mel2, defined as melanocyte stem cells, and Mel3, defined as mature melanocytes. Mel2 cells were prone to pyroptosis and necroptosis, while Mel3 cells were susceptible to oxidative stress, mitochondrial dysfunction, and ferroptosis. Compared with control mice, higher expression of CXCL16 on dendritic cells and of the CXCL16 ligand, CXCR6, on γδT cells were observed in leukoderma. Dendritic cells and natural killer T cells in the CUMS mouse spleen exhibited elevated levels of CXCL16 and CXCR6, respectively. Activation of the CXCL16-CXCR6 axis and a non-specific immune response in our CUMS model might imitate chronic mental stress-induced vitiligo in humans better than CD8 + cytotoxic T lymphocyte-mediated models.

**Conclusions:**

We discovered two melanocyte clusters with distinct fates and a pro-inflammatory micro-environment with CXCL16–CXCR6 axis activation of antigen-presenting cells and other innate immunocytes that might provide new insights into the pathogenesis of stress-induced vitiligo.

**Supplementary Information:**

The online version contains supplementary material available at 10.1186/s10020-025-01236-z.

## Introduction

Vitiligo is an immune-mediated inflammatory skin disorder with a prevalence of 0.5%–2%. It is characterised by depigmented macules and patches that result from a progressive loss of functional melanocytes in the skin and/or hair (Frisoli et al. 2020). Several factors have been put forward for the cause of melanocyte loss in vitiligo, including genetic defects, chemical exposure, oxidative stress, autoimmune dysregulation, and psychosocially stressful events (Ezzedine et al. 2015). Lai (Lai et al. 2017) and Bibeau (Bibeau et al. 2023) reported that more than half of patients with vitiligo suffered from stress (2078 of 3541 [58.7%]), and were more likely to have a psychosocial burden, such as anxiety (1019 of 3541 [28.8%]) and depression (866 of 3541 [24.5%]). According to data from the Health Improvement Network database, Vallerand found the risk for vitiligo among those with major depressive disorder (MDD) (MDD cohort, 532 of 405,397) was 64% higher than those without MDD (referent cohort, 6644 of 5,739,048) (After adjusting covariates, Hazard Radio = 1.64, 95% Confidence Interval: 1.43–1.87) (Vallerand et al. 2019). These findings indicated that psychosocial stressors are closely associated with vitiligo onset and flare-ups; however, the underlying mechanism is not known.

To date, due to the rapid reproduction rates, and convenient genetic manipulation, several mouse models are often used in vitiligo research, and the main mouse models of vitiligo can generally be divided into three categories: spontaneous models, transgenic mouse models, and induced models (Wu et al. 2024). Spontaneous models include the Mivit mouse and Blt mouse, which are inconvenient to access and unstable (Aaron et al. 1986; Johnson et al. 1992). Transgenic mouse models, including the FH, h3 T-A2, and Vitesse mouse models, have targeted melanocyte-related antigens, such as pre-melanosome protein (PMEL), tyrosinase-related protein (TRP), or tyrosinase (TYR)(Eby et al. 2014; Gregg et al. 2010; Mehrotra et al. 2012), and are inclined to elicit a cytotoxic CD8 + T lymphocytes immune response. Induced mouse models can be classified into endogenous and exogenous. Endogenous models often depend on vaccination or melanoma transplantation into Treg-depleted C57BL/6 J mice (Xu et al. 2022), which induces post-melanoma leukoderma. And monobenzone, hydrogen peroxide, hydroquinone, and Rhododenol are conventional exogenous chemical toxins that induce depigmentation models of depigmentation (Erdoğan et al. 2022; Wu and Wang 2024; Zang et al. 2022).

Considering the vital role of psychiatric factors in vitiligo, we designed and optimized a chronic unpredictable mild stimulation (CUMS) model to imitate the natural process of vitiligo onset under mental stress (Markov and Novosadova 2022). Our CUMS model exhibited obvious depigmentation and leukotrichia on the dorsal and tail skin. We investigated the immune microenvironment in CUMS mice and revealed striking similarities with human vitiligo. Our mouse model is a promising tool for exploring the pathogenesis of stress-associated vitiligo.

## Methods

### Establishment of a Chronic Unpredictable Mild Stress (CUMS) mice model

Our study examined male mice because male animals exhibited less variability in phenotype. It is unknown whether the findings are relevant for female mice. All animal studies were carried out in accordance with the guidelines and regulations of the Institutional Animal Care and Use Committee (IACUC) at Nanjing Medical University (IACUC-2307036). Five-week-old male C57BL/6 J mice weighing 18–22 g (g) were purchased from the Jiangsu Animal Experimental Center of Medical and Pharmaceutical Research. Mice (*n* = 40) were randomly divided into a control group (*n* = 20) and a CUMS group (*n* = 20), and all were maintained in a specific pathogen-free (SPF) environment, with free access to food and water (12 h (h) light/dark cycle at 20–22 degree centigrade (℃), humidity 45 ± 5%). Before the CUMS treatment, all the dorsal skin of both CUMS group and the control group were depilated simultaneously and were acclimated to the animal facility with a chow diet for one week.

CUMS group mice were given eleven stimuli for 12 weeks. The stimuli were cage swap (24 h), cage tilt (at an angel 45°, 6 h), cage wobble (20 min), wet litter (250 mL of water on the bed litter, 24 h), confinement (in a 50-ml centrifuge tube, 4 h), cold water swimming (4℃, 5 min), water bath (empty cage with 250 ml water, 1 h), predator sound (owl prey cries, 80Db 1 h), predator odor (2,4,5-trimethyl-3-thiazoline (TMT) to imitate a fox, stroboscopic lighting (rapid light/dark change, 1 h), tail suspension (20 cm off the ground, 5 min). The extreme stimuli of food deprivation (12 h) and water deprivation (12 h) were not used. The mice were modeled by alternating three or four stimuli each day, and each stimulus was used discontinuously for three days so that the mice could not predict and adapt to the stimulus.

The body weights of mice were measured once a week throughout the study. At week 12, approximately 60% of the mice in the CUMS group showed depigmentation and leukotrichia on the dorsal and tail skin (these mice were used in subsequent experiments). Full-length photographs were taken using a Canon D30 camera, and dermatoscopy images with Wood’s lighting were captured by device 3Gen DermLite DL4.

Behavior experiments, including Forced Swimming Test (FST), Tail Suspension Test (TST), Open Field Test (OFT), and Sucrose Preference Test (SPT) were performed at week 12. After behavioral evaluation, mice were euthanized, and skin, spleen, and serum were collected for histological evaluation and metabolic profiling.

### Single-cell sample collection

Melanocytes are present all over the human body skin, however, there is little difference in mice. Melanocytes are few in the epidermis of dorsal skin but exist in the hair follicles during anagen, while melanocytes exist in both the epidermis and hair follicles of mouse tail skin (Urtatiz et al. 2021). Although we observed the hair pigmentation disorder both in dorsal skin and tail skin by Dermatoscope with Wood’s lamp (hair and plaques with blue-white fluorescence in the red box indicated pigmentation disorder), the white patches were much more obvious in tail skin, therefore, we finally decided to choose the tail skin for Sc-Seq. Skin and spleen samples were processed as follows: Blunt dissection was used to isolate the intact tail skin, and the epidermis and dermis were separated using Dispase II (Sigma). The minced epidermis was further digested with 0.25% Trypsin–EDTA (Gibco) for 30 min and filtered through a 70-μm cell strainer (Falcon). The dermis was digested with 1.5 mg/mL Collagenase P (Sigma-Aldrich) and 50 U/mL DNase I (Sigma-Aldrich) at 37 °C for 1 h and also filtered through a 70-μm cell strainer (Falcon).

ScRNA-seq data from vitiligo patients and healthy controls were downloaded from the Genome Sequence Archive (GSA) with accession number PRJCA006797 to validate the findings observed in mice.

### scRNA-seq library preparation

Single-cell barcoding and library construction were performed using the BD Rhapsody system. Single-cell capture was achieved by randomly distributing the single-cell suspension across more than 200,000 microwells using a limited dilution method. Beads with oligonucleotide barcodes were added to saturation to pair beads with the cells in the microwells. The cells were lysed in the microwells, allowing the hybridization of mRNA molecules with barcode-capturing oligonucleotides on the beads. Beads were then collected into a tube for reverse transcription and ExoI digestion. After cDNA synthesis, each cDNA molecule was labeled at the 5′ end (i.e., the 3′ end of the mRNA transcript) with a unique molecular identifier (UMI) and a cell barcode to indicate its cell of origin. Whole-transcriptome libraries were prepared using the BD Rhapsody single-cell whole transcriptome amplification (WTA) workflow, including random priming extension (RPE), RPE amplification PCR, and WTA index PCR. Libraries were quantified using a High Sensitivity DNA chip on a Bioanalyzer 2200 (Agilent) and the Qubit High Sensitivity DNA Assay (Thermo Fisher Scientific). Sequencing was performed on an Illumina sequencer (Illumina, San Diego, California) with a 150-bp paired-end run. Adapter sequences were filtered out, and low-quality reads were removed using fastp with default parameters to obtain clean data. UMI-tools were employed for single-cell transcriptome analysis to identify cell barcode whitelists. Clean data based on UMIs were mapped to the mouse genome (Ensembl release 100) using STAR with custom parameters from the UMI-tools standard pipeline to obtain UMI counts for each sample.

### scRNA-seq data analysis

The single-cell RNA sequencing (scRNA-seq) expression matrix was subjected to analysis using Seurat (version 4.3.1). Cells were filtered based on a gene count range of 250 to 5000, a total number of unique molecular identifiers (UMIs) exceeding 500, and a percentage of mitochondrial gene UMIs below 20%. Following the exclusion of low-quality and contaminated lineage cells, scaled data were derived through regression of UMI counts and the mitochondrial rate percentage for each sample. A principal component analysis (PCA) matrix was established utilizing the scaled data and the top 2,000 highly variable genes. Subsequently, a nearest neighbor graph was constructed with the FindNeighbors function, and cells were clustered using the FindClusters function, with a resolution parameter set to 0.8. The first 10 principal components were employed to generate two-dimensional embeddings via uniform manifold approximation and projection (UMAP) and t-distributed stochastic neighbor embedding (t-SNE). Cell type annotations were assigned based on the expression profiles of established canonical marker genes. Differentially expressed genes (DEGs) for each cell subcluster were identified using the “FindAllMarkers” function and the Wilcox rank sum test algorithm, adhering to the following criteria: 1. log2 FC > 0.25; 2. corrected *p*-value < 0.05; 3. min.pct > 0.1.

### Ro/e Analysis

Ro/e analysis measures the enrichment of cell clusters in different tissues by using the ratio of observed to expected cell numbers. The formula is expressed as Ro/e_ij_ = O_ij_/E_ij_. In this equation, O_ij_ refers to the actual number of type i cells observed in pathogenesis j, while E_ij_ represents the expected count, calculated as E_ij_ = (T_i_ × P_j_)/T. Here, T_i_ denotes the total number of type i cells across all pathogenic conditions, P_j_ indicates the total number of cells in pathogenesis j, and T is the overall number of cells analyzed. Ro/e > 1 indicates that the given cell population in a specific tissue is observed more frequently than expected by random chance, signifying enrichment. Conversely, a ro/e < 1 indicates depletion, meaning that the cell population in a specific tissue is observed less frequently than expected by random chance (Zhang et al. 2018).

### Pathway enrichment analysis

Differentially expressed genes with |logFC|> 0.5 and adjusted *P*-value < 0.05 were used for Gene Ontology enrichment analysis. The gene set used was from the MSigDB database. To evaluate melanocyte activation, cell death-related pathways, T cell-related functions, macrophage polarisation, and dendritic cell antigen presentation, enrichment scores were calculated for each cell type or group using AUCell (version 1.22.0) or GSVA (version 1.50.0).

### Transcription factor regulon activity analysis

Transcriptional regulatory networks in single cells were assessed using the SCENIC (version 0.12.1). Regulons for each transcription factor were constructed using motif datasets, and co-expressed genes for each transcription factor were computed using GENIE3. The Spearman correlation between transcription factors and potential targets was then calculated. Finally, regulon activity was analyzed using AUCell.

### Trajectory Analysis

Single-cell trajectories were determined using Monocle3 (version 1.3.5) (Qiu et al. 2017). The clusters were initially segmented into sizable, distinct sections utilizing the cluster_cells function. Subsequently, a principal graph was constructed within each section through the learn_graph function. These principal graphs on the UMAP delineated the differentiation pathways. Based on previous knowledge, certain cell subtypes were selected as the roots of the cell differentiation trajectories.

### Cell–cell Interaction

To explore cell–cell interactions among different cell types, the CellChat R package (version 1.5.0) (Qiu et al. 2017) was used to assess the significance of signaling pathways inferred from the differences in overall information flow between the CUMS and control groups. Enriched receptor-ligand interactions between different cell types are inferred based on the expression of the receptor by one cell type versus the expression of the corresponding ligand by another cell type. The netVisual_circle function was used to visualize the strength of the cell–cell communication networks from the target cell cluster to various cell clusters within the significant signaling pathways. The netVisual_bubble function and the netVisual_hierarchy function created bubble plots and hierarchical plots, representing significant ligand-receptor interactions between the target cell cluster and other clusters.

#### Detection of cytokines in serum

Interferon-gamma (IFNγ), interleukin 6 (IL6), C-X-C motif chemokine ligand 10 (CXCL10), and CXCL16 in sera were detected using commercial ELISA kits (MAISHA INDUSTRIES, MS-0182M2, MS-0163M2, MS-45188M2, MS-2015B) according to the manufacturer’s instructions. All samples were measured in duplicate.

#### Immunohistochemistry

Tissues were fixed with 10% formalin overnight, embedded in paraffin, and then sectioned at a thickness of 5-μm. Sections were then processed to remove paraffin and then hydrated in alcohol and phosphate-buffered saline. Sections were made permeable in 0.2% Triton X-100 (Sigma-Aldrich), then blocked with 10% normal goat serum (Sigma-Aldrich), and then incubated with primary antibodies overnight at 4 ℃. Sections were then incubated with appropriate fluorescence-labelled secondary antibodies for 1 h at room temperature. Nuclei were stained with DAPI. Antibodies used: DCT (Proteintech, Cat 13,095–1-AP, 1:250), c-Kit (Proteintech, Cat 18,696–1-AP, 1:250), Anti-MelanA (Abcam, ab187369, 1:100), Anti-Cytokeratin 15 (Abcam, ab52816, 1:100), CD34 (Servicebro, Cat GB15013, 1:50), GSDMD (Proteintech, Cat 20,770–1-AP, 1:100), NLRP3 (Proteintech, Cat 68,102–1-Ig, 1:200), CD3 (Servicebro, Cat GB151137, 1:500), TCR γ/δ (Biolegend, Cat 118,101, 1: 150), CXCR6 (Solarbio, Cat K011438P, 1:50), Anti-NKR-P1 C (Abcam, ab289542, 1:100), CD8 (Servicebro, Cat GB15068, 1:400), CD44 (Servicebro, Cat GB112054, 1:150), CD127 (Abmart, Cat TD6362, 1:50).

#### Multi-color Immunohistochemistry

Multi-colour immunohistochemistry was performed using a TSAPlus Fluorescent Staining Kit according to the manufacturer’s protocol (Servicebio, China). sequential application of different primary antibodies, a secondary horseradish peroxidase-conjugated antibody with tyramide signal amplification (TSA) at 1:100 in 1 × amplification diluent was added and incubated at room temperature for 10 min. The sections were microwave heat-treated after each TSA operation. Multispectral images were collected using a Pannoramic MIDI (3DHISTECH) automatic digital slide scanner with the fluorescence spectra captured at 460 nm, 520 nm, 570 nm, and 670 nm with identical exposure times. Images were analyzed using Caseviewer Image Analysis Software (Danjier, version 2.4).

#### Haematoxylin–eosin and Fontana-Masson Staining

Mouse tail skin was embedded in paraffin. The embedded tissue was cut into 4 μm-thick slices adhered to the slides and dried at 45℃ for 4 h. The sections were stained following the protocol of hematoxylin and eosin, and Fontana-Masson for melanin. Images were captured and analyzed with SlideViewer (version 2.6.0).

#### Quantification and statistical analysis

The details of the replicates for each experiment are listed in the respective figure legends. In brief, unless otherwise indicated, for most in vitro or ex vivo or in vivo studies, the data are pooled from or representative of two to four independent experiments and are shown as the mean ± SEM. Comparison among groups was assessed by Student’s t-test or one-way analysis of covariance (ANOVA), and correlations were analyzed with Pearson’s correlation analysis. All statistical analyses were performed with the statistical software GraphPad Prism and *p* values < 0.05 were considered statistically significant. Statistical significance is defined as ****p < 0.0001, ***p < 0.001, **p < 0.01, *p < 0.05, or ns (not significant).

#### Role of funders

This study was independently conducted by authors and the funders had no role in study design, collection, analysis, interpretation, manuscript writing, and submission.

## Results

### Characterization of a CUMS-induced hair and skin whitening mouse model

**Fig. 1:**
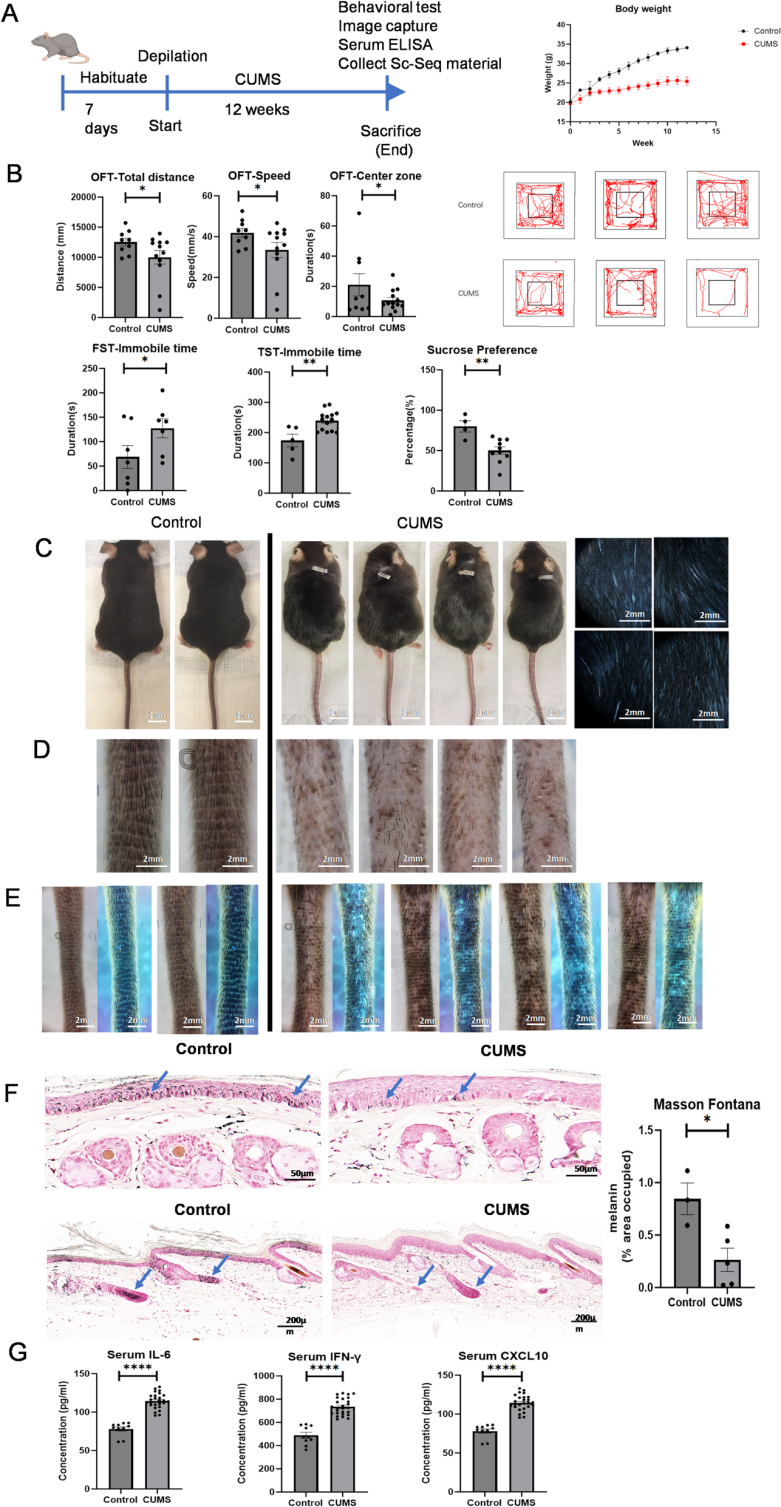
Optimization of Chronic Unpredictable Mild Stress (CUMS) induced hair and skin whitening mouse model. **A** Timeline of CUMS model and body weight change. **B** Behavioral test of CUMS, including FST, TST, SPT, and OFT with trajectory record. **C** Serum IFN-γ, IL-6, and CXCL10 detected by ELISA kit. **D** Full-length portraits taken by Canon D30 revealed that CUMS induced dorsal hair and tail skin to turn white **E**. Dermatoscope images were captured by device 3Gen DermLite DL4, **F** wood’s light photos of Control and CUMS group. **G** Melanin in epidermis and follicles of mouse tail skin. (Left are from control group, and right from CUMS. Fontana-Masson, HE stained × 400, scale bar = 50 μm. Data are from three independent experiments). Each sample is represented as one dot. *P*-values were calculated using two-sided Mann–Whitney U-test. *p < 0.05, ** *p* < 0.005 and *** *p* < 0.001. Abbreviations: FST, Forced Swimming Test; TST, Tail Suspension Test; SPT, Sucrose Preference Test; OFT, Open Field Test

Mental stress can result in depigmentation or inhibition of hair growth (Zhang et al. 2024). We therefore generated a CUMS mouse model to observe melanin loss. A flow chart of the study is shown in Fig. 1A. Body weight reflects the stress state of mice. As shown in Fig. 1A, the body weight of mice in the control group increased steadily over time, while it was lower in the CUMS group (*p* < 0.001). Behavioral tests can reflect a stressed state. In the open field test, CUMS mice displayed significantly reduced total distance traveled (*p* < 0.001), speed of movement (*p* < 0.001), and time spent in the center zone (*p* < 0.05) (Fig. 1B), indicating a significant reduction in spontaneous activity. Similarly, CUMS significantly increased the total immobility time in the forced swimming test and the tail suspension test (*p* < 0.05) (Fig. 1B), indicating that CUMS can lead to depression-like behaviour in mice.

To evaluate the impact of CUMS on melanin synthesis, we photographed mice using a Canon camera and a dermatoscope with Wood’s lamp. Compared with control mice, CUMS mice exhibited more depigmentation in the dorsal skin and tail (Fig. 1C–E). Haematoxylin & eosin staining combined with Fontana-Masson staining revealed melanin particles in the epidermis and hair follicles of the tail skin in CUMS mice (Fig. 1F). ELISAs showed that inflammatory cytokines associated with vitiligo, such as IFNγ, IL6, and CXCL10 were significantly elevated in CUMS serum (Fig. 1G). Together, these findings indicate that CUMS impeded melanin synthesis.

### Sequencing of single skin cells from control and CUMS mice reveals diversity in skin cell populations

**Fig. 2:**
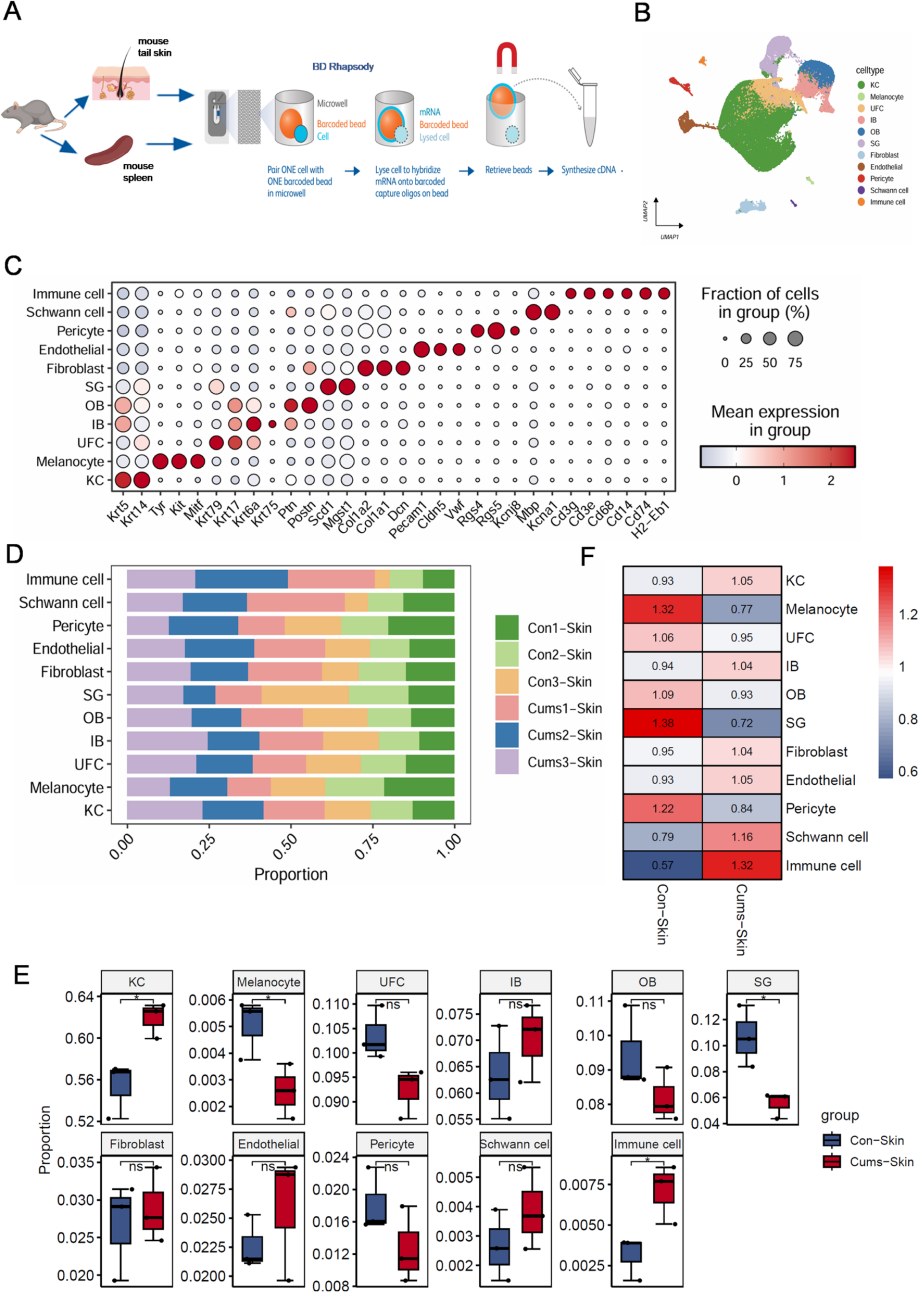
scRNAseq identifies well-defined cell populations in CUMS mice skin. **A** Flow chart of single cell sequencing. **B** Uniform Manifold Approximation and Projection (UMAP) plot for 14,211 high quality single cell transcriptomes from mouse skin with Control (*n* = 3) and CUMS (*n* = 3), revealing 11 different cell populations. **C** Dot plot displaying top expressed genes in each cell population compared to the other cell populations identified in skin. **D** Fractions of cell populations identified in the skin of Control and CUMS. **E** Boxplot of the distribution of eleven selected cell populations identified in the skin of Control and CUMS. **F** Tissue preference of each cluster estimated by the Ro/e index, which Ro/e > 1 suggests enriched, and Ro/e < 1 suggests depleted. Abbreviations: IB, inner bulge; KC, keratinocytes; OB, outer bulge; SG, Sebaceous gland; UFC, upper hair follicle cells (infundibulum)

We conducted single-cell RNA sequencing (scRNA-seq) on mouse tail skin using the BD Rhapsody System (Fig. 2A). This generated transcription profiles of 72,011 cells. The data from multiple sequencing runs were integrated into a unified Uniform Manifold Approximation and Projection (UMAP), which identified 11 different cell clusters (Fig. 2B). These included keratinocytes (*Krt15*, *Krt14*), fibroblasts (*Col1a2*, *Col1a1*, *Dcn*), melanocytes (*Tyr*, *Kit*, *Mitf*), upper hair follicle cells (infundibulum, *Krt17*, *Krt79*, *Krt6a*), immune cells (*Cd3*, *Cd68*, *Cd14*, *Cd74*, *H2-Eb1*), endothelial cells (*Pecam1*, *Cldn5*, *Vwf*), sebaceous gland cells (*Scd1*, *Mgst1*), outer bulge cells (*Postn*, *Ptn*, *Krt5*, *Krt17*), inner bulge cells (*Krt6a*, *Krt75*, *Krt5*, *Krt17*, *Ptn*), Schwann cells (*Mbp*, *Kcna1*), and pericytes (*Rgs4*, *Rgs5*, *Kcnj8*). Classical marker genes were highly expressed in all 15 cell types (Fig. 2C). All cell subtypes were present in both the CUMS and control groups (Fig. 2D). A boxplot of cell proportions showed a decrease in melanocytes and an increase in immune cells in the tail skin of the CUMS group compared with the control group (Fig. 2E). A heatmap based on the ro/e index displayed a consistent distributional preference among the cells (Fig. 2F).

### Impaired maturation and senescence of hair follicle melanocyte stem cells in CUMS mice skin

**Fig. 3:**
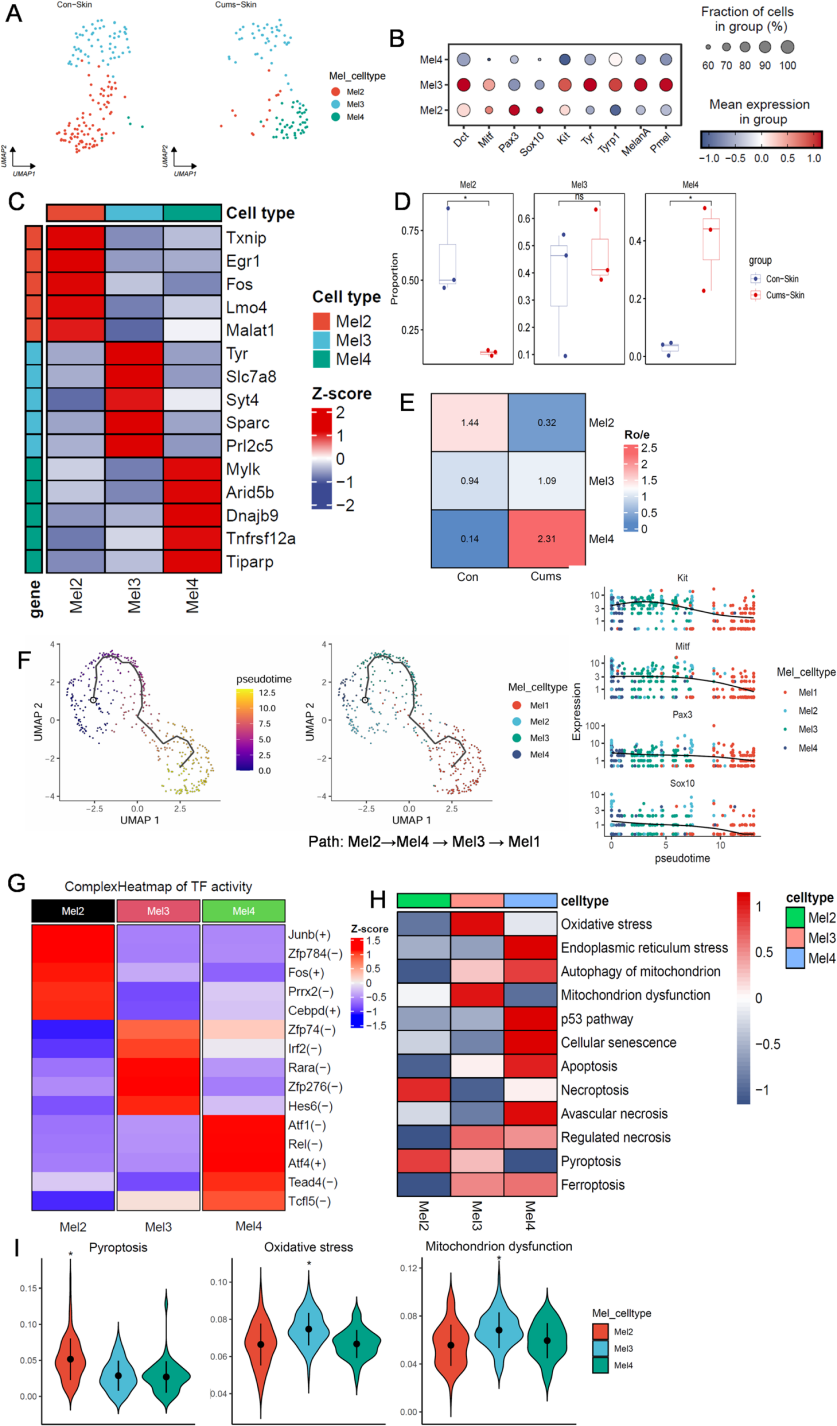
Characterization of the melanocyte population in mouse skin by scRNASeq. **A** UMAP plot for melanocyte cluster, revealing three different cell populations. **B** Dot plot displaying top expressed genes in each melanocyte cluster. C. Heatmap of Top 5 marker gene expression of three melanocyte clusters. D. Boxplot of the distribution of three selected cell populations identified in melanocyte clusters. E. Ro/e index of each cluster. Ro/e > 1 suggests enriched, and Ro/e < 1 suggests depleted. F. The inferred pseudo temporal trajectories of all cells using Monocle 3. Cells were colored by the inferred pseudo time. G. Heatmap of transcription factor activity of each cluster. H. Gene Set Variation Analysis (GSVA) for cell death modes and related pathways in each melanocyte cluster. I. Violin blots of pyroptosis, Oxidative stress, and mitochondrion disfunction. Each sample is represented as one dot. *P*-values were calculated using two-sided Mann–Whitney U-test. *p < 0.05

Clustering analysis defined four melanocyte sub-clusters, Mel1–Mel4 (Figure S2a). The top five marker genes heatmap for each sub-cluster revealed that the Mel1 cluster expressed keratinocyte-related markers, such as Krt14, Krt5, Krtdap, Krt1, and Krt10, which was quite different from the other three melanocytes sub-clusters (Figure S2b,c). Moreover, the GO enrichment analysis of Mel1 cells also showed Mel1 cells were enriched in cytoplasmic translation, translation at synapse, translation at pre-synapse, translation at post-synapse (Figure S2 d), which we presumed that these represented keratinocytes containing transferred melanin granules, and were therefore excluded from the melanocyte sub-clusters (Fig. 3A).

**Fig. 4:**
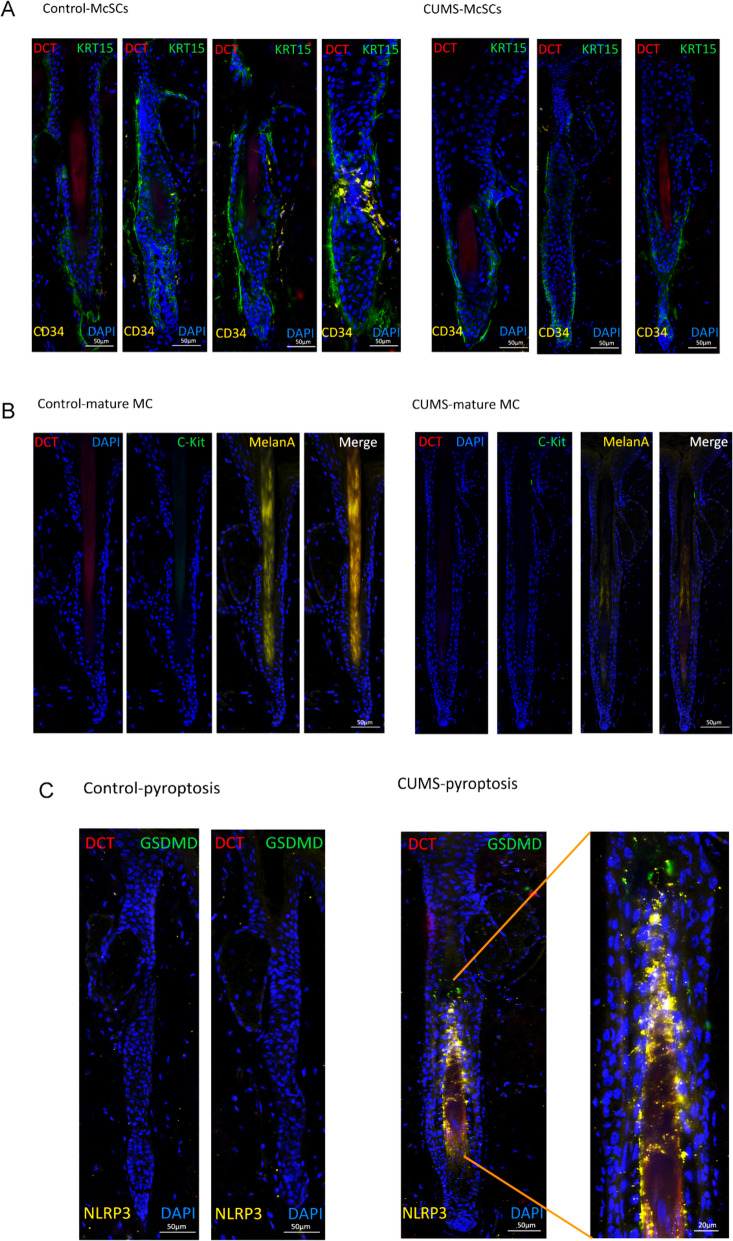
Immunofluorescence of Melanocytes from mouse tail skin. **A** Mel2 represented McSCs reduced in CUMS. DAPI (Blue), DCT (red), KRT15 (Green), CD34 (Yellow). **B** Mel3 represented Mature Melanocytes reduced in CUMS. DAPI (Blue), DCT (red), C-Kit (Green), MelanA (Yellow). **C** Pyroptosis was increased in CUMS. DAPI (Blue), DCT (red), GSDMD (Green), NLRP3 (Yellow). scale bar = 50 μm, Partial enlarged view scale bar = 20 μm. Data are from three independent experiments. Abbreviations: MC, melanocyte; McSC, melanocyte stem cell

Both cell proportions and the ro/e index showed a significantly decreased Mel2 subcluster in the CUMS group (Fig. 3D,E). Mel2 expressed high levels of melanocyte stem cell (McSC) markers (*Dct*, *Mitf*, *Pax3*, *Sox10*, and *Kit*), but lacked expression of melanin synthesis and proliferation markers (*Tyr*, *Tyrp1*, *MelanA*, and *Pmel*) (Fig. 3B,C). Consequently, we identified the Mel2 cluster as McSCs, while the Mel3 cluster represented mature melanocytes. To study the differentiation of melanocytes, we arranged the cells in pseudo-time order using monocle3 and observed a trajectory from Mel2 to Mel3 differentiation. During the differentiation process, a gradual reduction of McSC markers such as Mitf, Pax3, Sox10, and Kit was noted, further supporting the stem cell identity of Mel2 (Fig. 3F). Mel2 cells expressed high levels of genes such as *Txnip*, *Egr1*, and *Fos*, and also exhibited increased predicted transcription factor activity for *Junb*, *Fos*, *Prrx2.* Notably, *Fos* was enriched in Mel2 at both expression and predicted transcription factor activity levels (Fig. 3C, G). The transcription factor “Fos” is rapidly induced by stimuli such as growth factors, cytokines, and stress. “FOS family” proteins can form heterodimers with “JUN family” proteins to create the Activator Protein-1 complex, regulating gene expression of cell proliferation, differentiation, necroptosis, pyroptosis, and immune responses (Angel et al. 2001; Zhang et al. 2025). Enrichment analysis indicated that Mel2 cells were significantly enriched in processes related to pigmentation and pigment cell differentiation, while Mel3 cells were enriched in pigmentation and developmental pigmentation processes (Figure S2e,f). Heatmap of cell death showed that Mel2 cells were prone to pyroptosis and necroptosis, while Mel3 cells were more susceptible to oxidative stress, mitochondrial dysfunction, and ferroptosis (Fig. 3H,I). Immunohistochemistry confirmed the existence of the Mel2 and Mel3 clusters and also showed pyroptosis in the Mel2 cluster (Fig. 4).

**Fig. 5:**
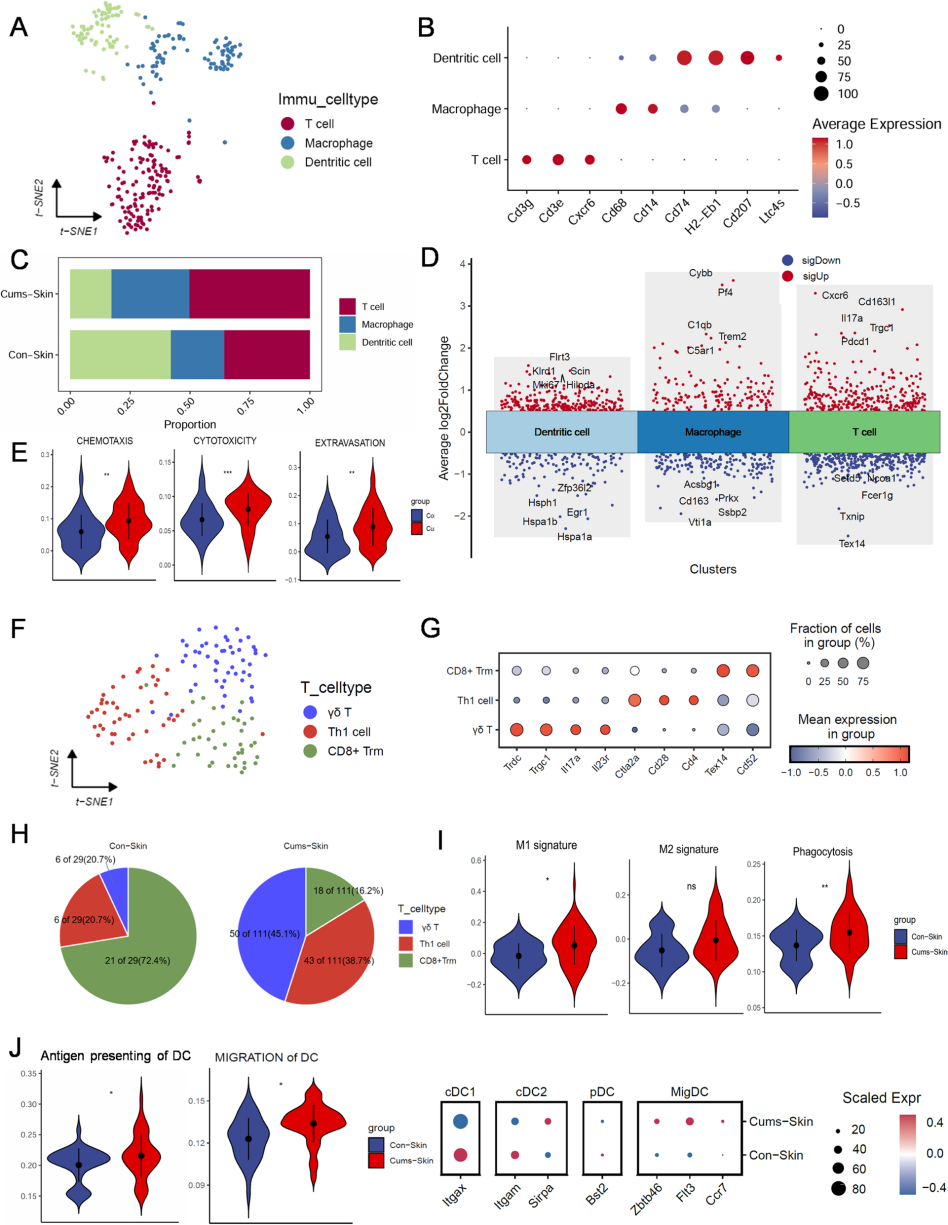
Characterization of the immune cells population in mouse skin by scRNASeq. **A** Immune cells t-Distributed stochastic neighbor embedding (t-SNE) plot. **B** Dot plot displaying top expressed genes in three immune cells clusters. **C** Bar plot showing the fractions of cell populations identified in mouse tail skin. **D** Muti-volcano plot of distribution of gene variation in CUMS and Control. **E** Dot plot displaying top expressed genes in three T cells clusters. **F** T cell function related violin plot. **G** Dot plot displaying top expressed genes in three T cells clusters. **H** Pie charts of proportions of three T cells clusters in CUMS and Control. **I** Violin plots of Macrophages signature. **J** Phagocytic function of Macrophages. **K** Antigen presentation scores of dendritic cells. *P*-values were calculated using two-sided Mann–Whitney U-test. *p < 0.05, ** *p* < 0.005 and *** *p* < 0.001. Abbreviations: DC, dendritic cells; Th1, helper T cells.; Trm, resident memory T cells

### Inflammatory endogenous environment of hair follicle cell populations in the tail skin of CUMS mice

The hair follicle niche in which McSCs are located plays an important role in maintaining their differentiation ability (Huang et al. 2024). Thus, we classified hair follicle cells into four clusters: hair follicle stem cells (HFSCs), upper hair follicle cells (infundibulum, UFCs), outer bulge cells (OBs), and inner bulge cells (IBs), with each cluster annotation shown in Figure S2a–d. McSCs are located in the bulge and hair germ (HG) area and are adjacent to HFSCs. We therefore focused on the reduced number of HFSCs in the CUMS group and found a significant increase in the expression of pro-inflammatory chemokines (*Il1, Il6, Il15, Ccl2, Cxcl12*) (Figure S3e). Overall, the expression of genes related to the γ-interferon pathway, such as *Ifngr1* and *Jak1*, was obviously increased in the HFSCs cluster (Figure S3f,g). These findings indicate that McSCs in the CUMS group are within an inflammatory internal environment.

### Keratinocyte and fibroblast populations in CUMS mouse tail skin concentrate on pro-inflammatory responses

In vitiligo lesions, stromal cells, especially keratinocytes and fibroblasts, are extensively involved in the inflammatory destruction of melanocytes by secreting various chemokines (Shi et al. 2024; Yang et al. 2023). The majority of cells sequenced in our scRNA-seq analysis constituted keratinocytes (42,408 cells). Sub-clustering analysis revealed significant proportional differences in the nine sub-clusters. These nine sub-clusters could be identified as four keratinocyte states based on previously reported expression profiles (Figure S4a). Hair follicle keratinocytes highly expressed *Krt15* and *Krt17*. Super-spinous keratinocytes expressed *Slurp1* and *Sprr1b*. Spinous keratinocytes specifically expressed early differentiation keratins (*Krt1* and *Krt10*). Basal keratinocytes highly expressed *Krt14*, *Mt2*, *Col17a1*, and *Dst* (Figure S4b,c). A deeper analysis revealed that all keratinocyte populations in the CUMS group were focused on inflammation and immune responses, including the interferon pathway, oxidative stress responses, and cytokine signalling (Figure S4 d).

Cutaneous fibroblasts (FCs) are heterogeneous and participate in multiple skin disorders(Plikus et al. 2021; Shi et al. 2024; Yang et al. 2023), including psoriasis, atopic dermatitis, and vitiligo (Cai et al. 2023; He et al. 2020; Xu et al. 2022). We explored the heterogeneity of FCs in the CUMS group and identified four subgroups. These subgroups were further categorised into Col1a1 + FCs (*Col1a1*, *Col3a1*, *CD34*, *Dcn*), and Pdgfa + FCs (*Pdgfa*, *Spon1*, *Serpine2*) for second-level analysis (Figure S4e,f). The Col1a1 + FCs cluster was significantly increased in CUMS mice and highly expressed the pro-inflammatory chemokines Il1b, Il6, Mmp2 and Ccl2 (Figure S4 g,h). Thus, this cluster represents pro-inflammatory fibroblasts that regulate the recruitment and organization of lymphocytes and myeloid cells.

### γδT initiates nonspecific immunity in the tail-depigmented skin region of CUMS mice

Next, we attempted to characterize the dynamics of the immune microenvironment in CUMS mouse tail skin. Immune cells in mouse tail skin were categorised into three clusters: T cells (*Cg3 g*, *Cd3e*, *Cxcr6*), Macrophages (*Cd68*, *Cd14*), and Dendritic cells (*Cd74*, *H2-Eb1*, *Cd207*, *Ltc4 s*). Compared with the control group, a t-distributed stochastic neighbor embedding (t-SNE) plot, and bar charts showed CUMS mice to have increased proportions of T cells and macrophages among immune cells (Fig. 5 A-D). We then evaluated the function of T cells and found that processes such as chemotaxis, cytotoxicity, and extravasation were all predicted to be enhanced in the CUMS group (Fig. 5E). Further analysis of T cells revealed three sub-clusters: γδT cells (*Trdc*, *Trgc1*, *Tob2*), Th1 cells (*Ctla2a*, *Cd28*, *Cd4*), and CD8 + resident memory T cells (CD8 + Trm) (*Tex14*, *Cd52*) (Fig. 5F,G). Dramatic increases in the numbers and proportion of γδT cells and Th1 cells were observed in CUMS mice compared with control mice, while numbers of CD8 + Trm remained unchanged, and only a decrease in its relative proportion due to the increase in γδT in the CUMS group (Fig. 5H).

Macrophages in the CUMS group expressed high levels of inflammatory factor genes, such as *Tnf*, *Ccl2*, *Ccl3*, *Ccl4*, and *Ccl22*, and were more inclined to an M1 pro-inflammatory phenotype with enhanced phagocytic function. (Fig. 5I). Dendritic cells in the CUMS group showed enhanced migration capacity with higher expression of migratory DC (migDC) genes, such as *Zbtb46*, *Flt3,* and *Ccr7*. Antigen-presentation scores were also increased (Fig. 5J).

**Fig. 6:**
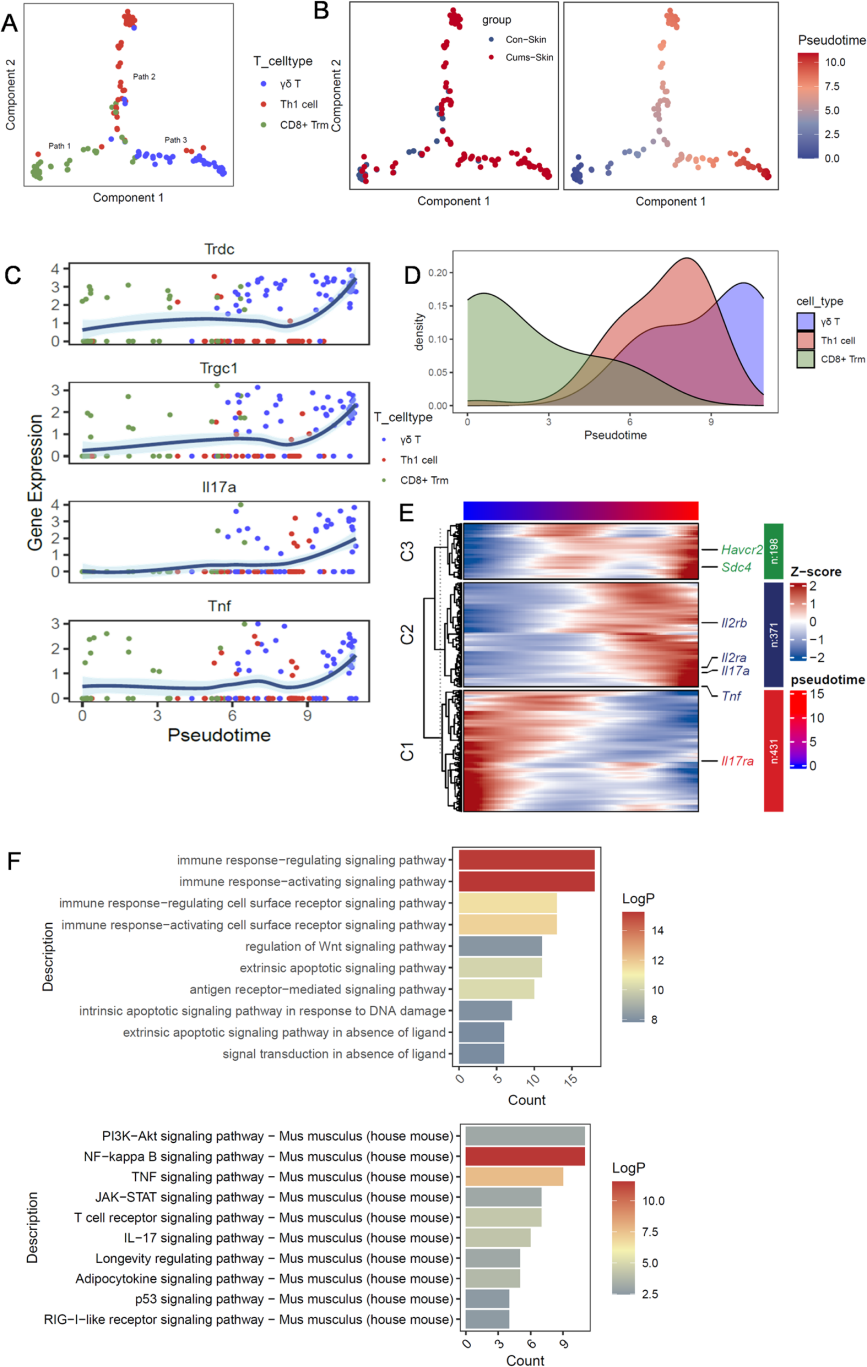
Characterization of the γδT cells population in mouse skin. **A**-**B** The inferred pseudo temporal trajectories of all T cells in skin using Monocle 3. Cells were colored by the inferred pseudo time. **C** Genes expression dynamics of Trdc, Trgc1, Il17a and Tnf of different T cells clusters, along the inferred pseudo-time axis. **D** Cumulative-density plot of different clusters of T cells. **E** The pseudo-time heat map of T cell proliferation genes and inflammatory genes during the occurrence of CUMS. **F** GO and KEGG enrichment analysis of γδT cells. Abbreviations: Th1, helper T cells; Trm, resident memory T cells

To fully understand the differentiation trajectory of T cells in the skin of CUMS mice, we used monocle analysis to reveal three major T cell pathways. Path 1 started with CD8 + Trm, and Paths 2 and 3 ended with Th1 and γδT states, respectively. CUMS caused gradual activation with the change in pseudo-time (Fig. 6A,B). We found that the cumulative γδT cell density increased gradually with pseudo-time, which was supported by the expression dynamics of γδT signature genes along the inferred pseudo-time axis (Fig. 6C,D). The pseudo-time heatmap revealed that expression of T cell proliferation genes, such as *Il2ra* and *Il2rb*, and inflammatory factor genes, such as *Il17a* and *Tnf*, gradually increased with CUMS exposure (Fig. 6E). In addition, GO and KEGG enrichment analyses showed that γδT cells were enriched in immune response activation, such as activation of NF-KB, TNF and JAK-STAT pathways (Fig. 6F). Together, these results indicate activation of innate immunity in depigmented skin and potential initiation of specific immunity characterised by enhanced migratory capacity of antigen-presenting cells.

### Ligand-receptor analysis reveals cell type-specific networks in CUMS mouse skin

**Fig. 7:**
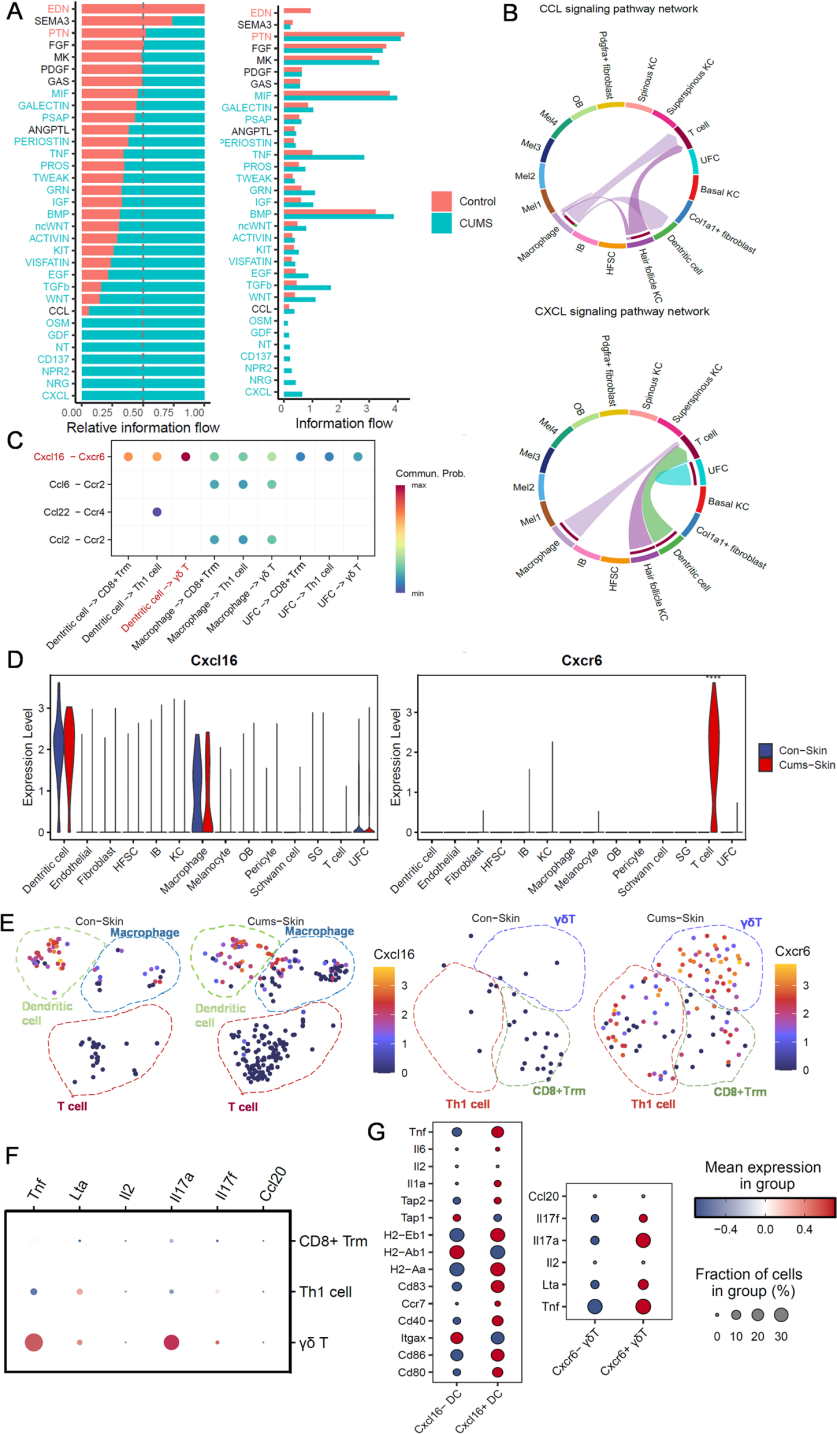
Cellchat of the immune cells with other cells from the mouse skin. **A** Significant signaling pathways were ranked based on differences in the overall information flow within the inferred networks between CUMS and Control skin scRNA-seq dataset. **B** Network visual chord diagram showing the CXCL and CCL signaling pathways ranked high in CUMS. **C** Comparison of the multiple ligand–receptor pairs among different cells. **D** Violin plots displaying the vary expression of CXCL16 and CXCR6 in all cell clusters at the mRNA level. **E** The feature plots showing CXCL16 and CXCR6 mRNA expression within total skin cells between CUMS and Control. **F** Dot plot showing different cytokines in each T cell cluster of skin. **G** Dot plot showing cytokines of Cxcl16-negative DC and Cxcl16-positive DC; cytokines of Cxcr6-negative γδT and Cxcr6-positive γδT. *P*-values were computed from one-sided permutation test (CellChat 1.5.0). *p < 0.05, ** *p* < 0.005 and *** *p* < 0.001. Abbreviations: DC, dendritic cells; HFSC, hair follicle stem cell; IB, inner bulge; KC, keratinocytes; OB, outer bulge; SG, Sebaceous gland; Th1, helper T cells; Trm, resident memory T cells; UFC, upper hair follicle cells (infundibulum)

We employed the CellChat package to elucidate the complex relationship between different clusters. By comparing the overall communication probability between the control and CUMS groups, we identified 33 enriched signalling pathways, with eight pathways being more prevalent in the CUMS group, including CXCL, NRG, NPR2, CD137, NT, GDF, OSM, and CCL (Fig. 7A). These pathways may therefore contribute to the progression of vitiligo. The CXCL and CCL signaling pathways in CUMS mice ranked highly in network visual chord diagrams (Fig. 7B). CXCL16-CXCR6 is the most significant ligand-receptor pair in CXCL signalling (Fig. 7C); therefore, we further examined the expression of these chemokines in all cell clusters at the mRNA level (Fig. 7D). Dendritic cells expressed significantly higher amounts of *Cxcl16*, and T cells, especially γδT cells, expressed *Cxcr6* according to the gene distribution UMAP (Fig. 7E). *Cxcl16*-positive DCs expressed more genes related to differentiation and mutation, like *Cd83, Cd80, CD86*, and *Cxcr6*-positive γδT cells expressed more pro-inflammatory cytokines such as *Il17, Il2, Tnf* (Fig. 7F,G). These results indicate that the CXCL16-CXCR6 ligand-receptor may participate in the immune infiltration. Detailed Cellchat information of the different cells with the Mel2 and Mel3 clusters is presented in Figure S5.

### Subsequent activation of nonspecific and specific immunity in the spleen of CUMS mice

**Fig. 8:**
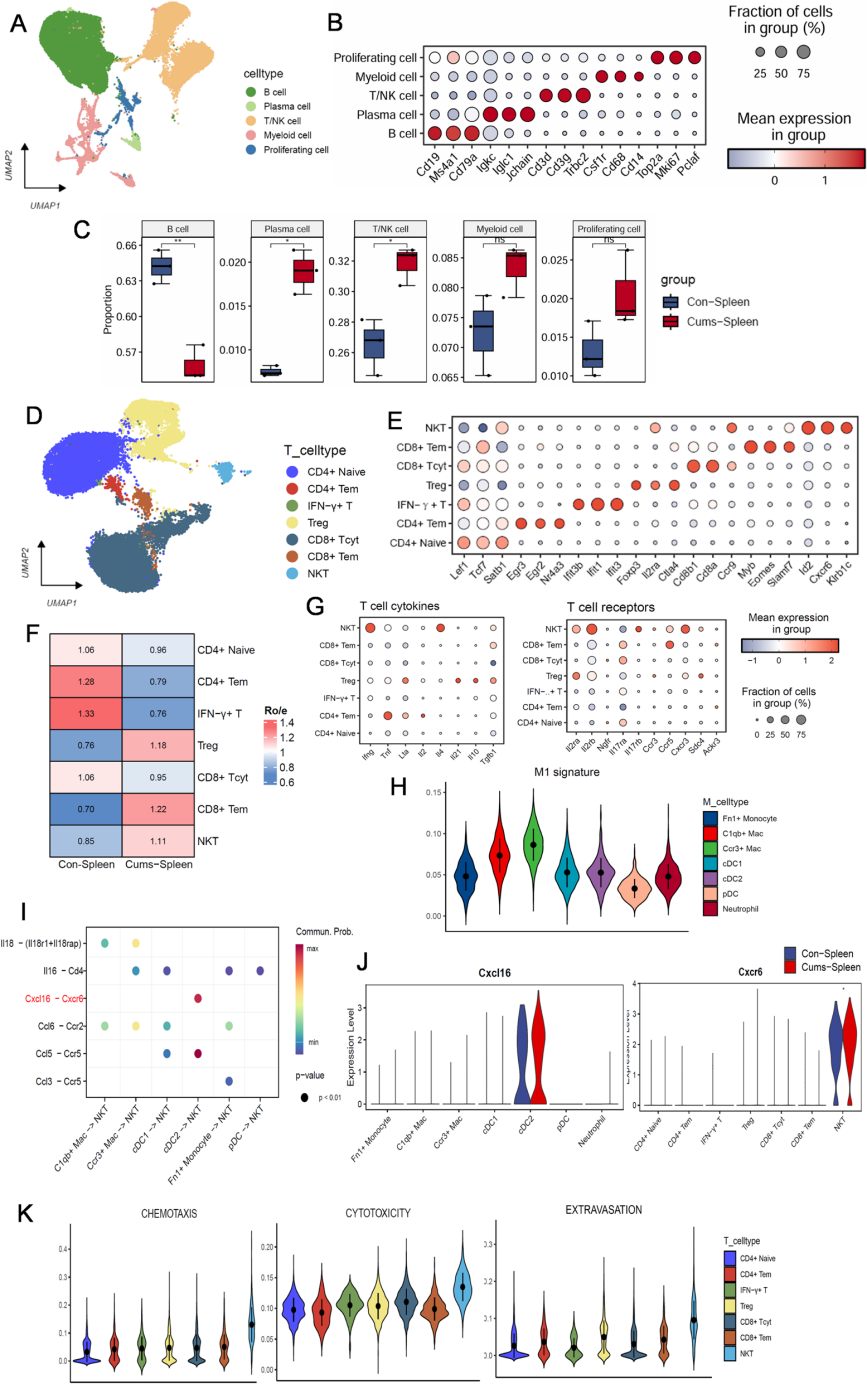
Characterization of the immune cells population in mouse spleen by scRNASeq. **A** UMAP of immune cells in mouse spleen. **B** Dot plot displaying top expressed genes in five immune cells clusters. **C** Bar plot showing the fractions of cell populations identified in mouse spleen. **D** UMAP of T cells clusters. **E** Dot plot displaying top expressed genes in seven T cells clusters. **F** Ro/e index of each cluster. Ro/e > 1 suggests enriched, and Ro/e < 1 suggests depleted. **G** Dot plot showing different cytokines and receptors in each T cell cluster. **H** Violin plots of Macrophages signature. **I** Comparison of the multiple ligand–receptor pairs among NKT with other cells. **J** Violin plots displaying the vary expression of CXCL16 and CXCR6 in all T cell clusters at the mRNA level in CUMS and Control. **K** NKT cell function related violin plot. *P*-values were computed from one-sided permutation test (CellChat 1.5.0). *p < 0.05, Abbreviations: DC, dendritic cells; Mac, macrophages; NKT, natural killer T cells; Tem, effector memory T cells; Tcyt, cytotoxic T cells; Treg, regulatory T cells

To investigate whether the migrating dendritic cells in the skin of CUMS mice affect the immune function of the spleen, we performed scRNA-seq on the spleen. After quality control, scRNA-seq data from mouse spleen were classified into five clusters: B cells (*Cd19*, *Ms4a1*, *Cd79a*), T/Natural killer (NK) cells (*Cd3*, *Trbc2*), Plasma cells (*Igkc*, *Iglc1*, *Jchain*), Myeloid cells (*Csf1r*, *Cd68*, *Cd14*), and Proliferating cells (*Top2a*, *Mki67*, *Pclaf*) (Fig. 8A,B). A notable increase in the proportion of T/NK cells was identified in CUMS spleen compared with control spleen (Fig. 8C). Subsequently, secondary analysis isolated seven sub-groups: CD4 + naive T cells (*Lef1*, *Tcf7*, *Satb1*), CD4 + effector memory T cells (*Egr3*, *Egr2*, *Nr4a3*), IFN-γ + T cells (*Ifit3b*, *Ifit1*, *Ifit3*), regulatory T cells (*Foxp3*, *Il2ra*, *Ctla4*), CD8 + cytotoxic T cells (*Cd8b1*, *Cd8a*, *Ccr9*), CD8 + effector memory T cells (*Myb*, *Eomes*, *Slamf7*), and NKT cells (*Id2*, *Cxcr6*, *Klrb1c*, *Ccr9*) (Fig. 8D,E). An increase in the proportion of CD8 + effector memory T cells, regulatory T cells, and NKT cells was identified (boxplot and Ro/e index, Fig. 8F,G). NKT cells expressed the highest levels of most pro-inflammatory cytokines, like *Ifng* (Fig. 8G). Therefore, we evaluated the functions of T cells, particularly NKT cells, which were predicted to be enhanced to varying degrees for chemotaxis, cytotoxicity, and extravasation (Fig. 8K).

To further explore whether NKT cells in the spleen of CUMS mice are activated by antigen-presenting cells, we conducted CellChat package analysis. We found Ccl5-Ccr5 and Cxcl16-Cxcr6 to be the most significant two pairs between cDC2 and NKT (Fig. 8I). Gene expression analysis showed that dendritic cells, more specifically the cDC2 cluster, expressed the highest level of *Cxcl16*. NKT cells expressed the highest level of *Cxcr6* in the mouse spleen (Fig. 8J). Immunohistochemistry confirmed the expression of *Cxcl16* in cDC2 and *Cxcr6* in NKT cells within the CUMS mouse spleen (Fig S6). Together, these results indicate the initiation of specific and non-specific immune responses represented by CD8 + Tem and NKT by the spleen after recognition of antigens and migration in cutaneous DCs.

### Highly cytotoxic γδT cells are also present in the marginal zones of vitiligo lesions

CD8 + T cell infiltration is a pathological feature of vitiligo lesions. However, previous studies have rarely reported the presence of γδT cells in the marginal zones of vitiligo lesions. Therefore, to search for evidence of the presence of γδT cells in skin lesions from patients with vitiligo, we downloaded a scRNA-seq dataset from the Genome Sequence Archive (accession number PRJCA006797), which includes normal skin samples from five healthy controls and marginal zone skin samples from 10 vitiligo patients.

**Fig. 9:**
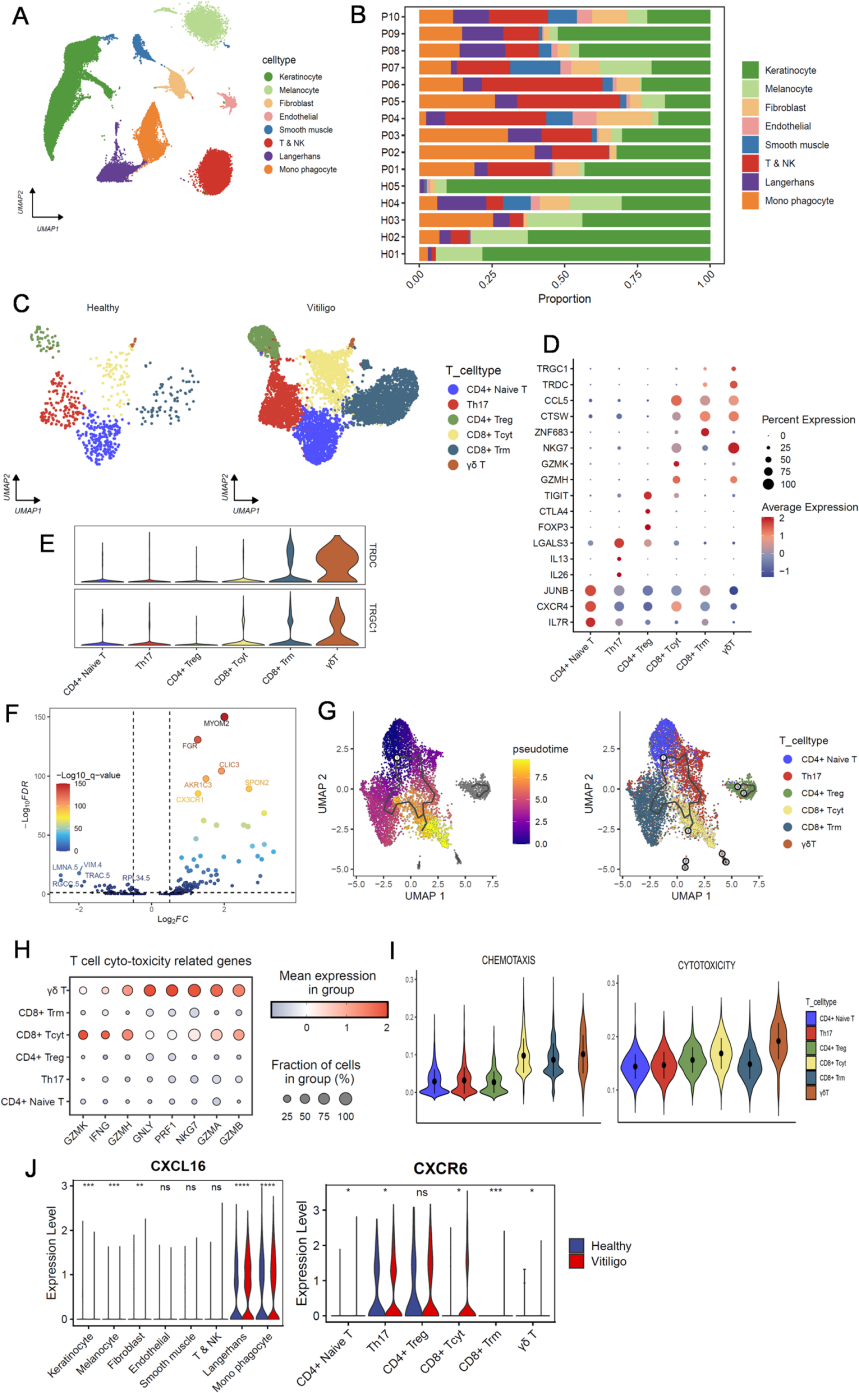
Characterization of the immune cells population in human skin by scRNASeq from dataset of GEA. **A** UMAP plot for 48,887 high quality single cell transcriptomes from dataset of GEA of human skin with Control (*n* = 5) and vitiligo (*n* = 10), revealing eight different cell populations. **B** Fractions of cell populations identified in the skin of Control and vitiligo. **C** UMAP of T cells clusters. **D** Dot plot displaying top expressed genes in each cell population compared to the other cell populations identified in skin. **E** Violin plot showing expression of γδT markers in six T cells clusters. **F** Volcano plot displaying the differential genes of γδT cells. **G** The inferred pseudo temporal trajectories of all T cells using Monocle 3. Cells were colored by the inferred pseudo time. **H** Dot plot of cytotoxicity function of all T cells. **I** Violin plots of chemotaxis and cytotoxicity function of all T cells. J. Violin plots displaying the vary expression of CXCL16 and CXCR6 in all T cell clusters at the mRNA level in healthy and vitiligo. *P*-values were calculated using two-sided Mann–Whitney U-test. *p < 0.05, ** *p* < 0.005 and *** *p* < 0.001. Abbreviations: Th, helper T cells.; Trm, resident memory T cells; Tcyt, cytotoxic T cells; Treg, regulatory T cells

After quality control, transcriptomes of 48,887 cells were retrieved (Fig. 9A). An increased proportion of T cells was observed in vitiligo samples compared with control samples (Fig. 9B). The T cell clusters were extracted and further unbiased clustering identified six T cell sub-clusters: CD4 + naive T cells, Th17, CD4 + Tregs, CD8 + cytotoxic T cells (CD8 + Tcyt), CD8 + resident memory T cells (CD8 + Trm), and γδT cells. Consistent with our scRNA-seq study in CUMS mice, γδT cell numbers were increased in the marginal zones of vitiligo patients, although they accounted for a smaller proportion (Fig. 9C,D). Interestingly, CD8 + Trm cells also expressed γδT markers, indicating that CD8 + Trm cells may have acquired some functional characteristics of γδT cells, such as an early rapid immune response (Fig. 9E).

**Fig. 10:**
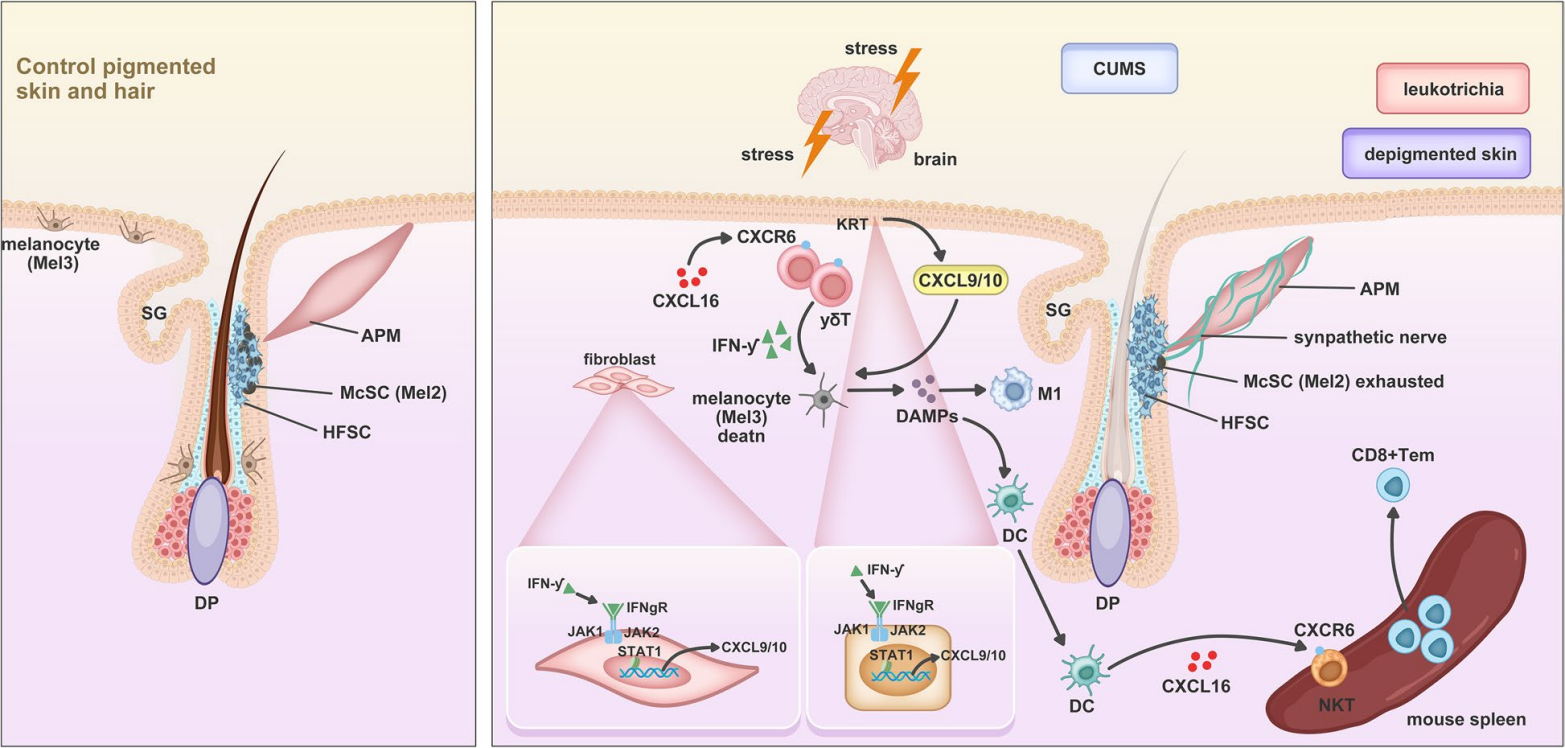
Hypothesis schematics of comprehensive immune microenvironment in an optimized CUMS-induced leukoderma and leukotrichia mouse model. Left is pigmented skin and hair in the control group, and the right is depigmented skin with leukotrichia in CUMS mice. CXCR6 + γδT cells, recruited by CXCL16, produce IFNγ that induce the secretion of CXCL9/10 by keratinocytes and fibroblasts, contributing to a pro-inflammatory microenvironment, resulting in direct or indirect injury of melanocytes in epidermis and follicles (Mel3) and McSCs (Mel2). Impaired melanocytes can release melanocyte-specific antigens and DAMPs which may transfer to nearby dendritic cells and induce their maturation into CXCL16-positive antigen-presenting cells (APCs). Next, these APCs migrate into peripheral lymph nodes and spleen to recruit CXCR6 + NKT cells to enhance inflammation. Abbreviations: APM, arrector pili muscle; CUMS, chronic unpredictable mild stress; DC, dendritic cells; DAMPs, damage-associated molecular patterns; HFSC, hair follicle stem cell; KC, keratinocytes; McSC, melanocyte stem cell; M1, macrophages M1 pro-inflammatory phenotype; NKT, natural killer T cells; SG, Sebaceous gland; Tem, effector memory T cells

Differential analysis indicated that γδT cells expressed high levels of certain genes, such as *MYOM2*, *FGR*, and *CX3 CR1* (Fig. 9F). In addition, γδT cells expressed high levels of T cell cytotoxic genes, such as *GZMA*, *GZMB*, and *NKG7*, and showed the highest cytotoxicity scores calculated by AUCell (version 1.22.0) (Fig. 9H,I). Pseudotime analysis revealed that γδT cells did not originate from other CD4 + or CD8 + T cells, displaying a distinct developmental state (Fig. 9G). Lastly, CXCL16-CXCR6, which plays an important role in cell communication in CUMS mice, was widely expressed across T cell sub-clusters and showed inter-group differences in γδT and CD8 + Trm cells (Figs. 9J and 10).

## Discussion

To investigate the depigmentation process under chronic mental stress, we established a CUMS mouse model with obvious leukotrichia and depigmentation in the tail, and with a notable decline in the number of melanin particles in the epidermis and hair follicles. These features are similar to those in other vitiligo mouse models.

Keratinocytes and FCs contribute to skin homeostasis and pathophysiology in vitiligo. Different subpopulations of keratinocytes and FCs recruit immune cells and influence nearby melanocytes by secreting chemokines (Shi and Liu 2024; Yang and Luan 2023). Recently, through scRNA-seq of vitiligo skin, Xu and colleagues identified a subpopulation of IFN-responsive fibroblasts that secrete IFNγ to recruit and activate CD8 + cytotoxic T cells, which are pivotal in shaping the vitiligo microenvironment (Xu et al. 2022). IFN-γ may activate the JAK-STAT signaling pathway, leading to the upregulation of cell cycle regulators such as p21 and p27, resulting in the senescence of melanocytes, potentially contributing to skin hypopigmentation. Secondly, IFNs can upregulate pro-apoptotic genes (BAX, FAS) and downregulate the anti-apoptotic genes (BCL-2), leading to increased melanocyte death. On the other hand, IFNs can affect melanocytes indirectly by producing oxidative stress associated ROS, releasing inflammatory cytokines to establish an inflammatory microenvironment, also activating immune cells (such as T cells, and NK cells), enhancing their ability to recognize and target melanocyte related markers (Frisoli et al. 2020; Xu et al. 2022). All above finally may contribute to the damage or dysfunction of skin melanocytes. Likewise, our CUMS model revealed a similar manifestation of keratinocytes and FCs, with elevated levels of IFNγ, IL1beta, IL6, MMP (matrix metalloproteinase) 2, and CXCL9/10, which might regulate the recruitment of immunocytes. This indicates almost the same pro-inflammatory micro-environment as found in human vitiligo.

Interestingly, we discovered unique defective melanocytes grouped into the Mel2 and Mel3 clusters in our CUMS model. The Mel2 cluster was identified as McSCs, which are prone to pyroptosis and necroptosis. In contrast, the Mel3 cluster represented mature melanocytes, which are inclined to undergo mitochondrial dysfunction and oxidative stress, resulting in regulated necrosis and ferroptosis. Consequently, activation of immune cells within the skin, particularly macrophages and T cells, including γδT cells and Th1 cells, is consistent with their role in the demise of mature melanocytes. Nonetheless, it remains unclear why McSCs exhibit a distinct fate.

To investigate the McSC niche, we focused on the organization of the McSC system, which resembles that of HFSCs. McSCs are situated in the bulge and hair germ (HG) area in telogen-phase hair follicles, and are surrounded by HF epithelial stem cells (bulge cells) and progenitor cells (HG cells), collectively forming the McSCs niche (Bisgaard et al. 2022; Huang and Zuo 2024). When the anagen growth phase begins, McSCs produce specialised melanocytes that move down into the hair bulb and basal layer of the epidermis, producing pigment for the hair and skin (Zhang and Chen 2024). Zhang and colleagues injected C57BL/6 J mice subcutaneously with resiniferatoxin (a capsaicin analogue) and reported increased numbers of unpigmented hairs on the dorsal (Zhang et al. 2020). They demonstrated an attractive hypothesis that sympathetic nerves might modulate McSCs activity, melanocyte migration, or pigment production, and extreme stress may result in hyperactivation of neuronal activities that overstimulate the pathway, eventually driving the migration and depletion of McSCs (Zhang and Ma 2020). Therefore, based on our observations, we hypothesized that despite the immune-privileged nature of hair follicles, which can protect them against immunotoxicity, the neighboring cells might discharge pro-inflammatory cytokines, such as *IFNGR1, IL1a, IL6, and IL5*, thereby triggering an inflammatory reaction. CUMS might generate the abnormal release of neurotransmitters, contributing to hyperactivation, migration, and depletion of McSCs. Further investigations are necessary to validate the mechanisms proposed here.

Yang (2023) employed high-resolution scRNA-seq to show that innate immune cells, such as DCs, NK/NKT cells, and γδT cells, are abnormally activated in the skin and blood of non-segmental vitiligo patients. NK/NKT cells have been implicated in the early development of vitiligo as potential initiators of T cell autoreactivity (Tulic et al. 2019; Zhou et al. 2012). Consistently, we found activation of γδT cells, indicating activation of innate immunity in depigmentation lesions. Compared with the control group, significantly higher levels of CXCL16 on DCs and of its ligand, CXCR6, on γδT cells were observed in leukoderma of CUMS mice. While in the spleen of CUMS mice, NKT cells exhibited elevated levels of CXCR6. CXCL16 levels are elevated in the peripheral blood of vitiligo patients and decline after treatment that effectively repigments lesional sites (Li et al. 2017). CXCL16 can be secreted from keratinocytes, dendritic cells, and even Langerhans cells, and functions as a chemokine by binding to its receptor CXCR6 to mediate homing of CD8 + T cells in vitiligo skin. CXCR6 is often expressed on a subset of type 1 polarised immune cells, including CD4 + and CD8 + T cells, γδT cells, natural killer T cells, and natural killer cells (Kang et al. 2024). The skin is equipped with a resident immune population that interacts with systemic immune cells. The spleen serves as a hub for immune cells, including T cells, B cells, DCs, and macrophages. The spleen can regulate skin immunity by activating immune cells migration to the skin with increased chemokines (such as CXCL16, CXCL10, CCL5, and CCL20) via the bloodstream, producing cytokine (such as IFN-γ, IL-6) affecting keratinocytes, fibroblasts, and immune cells, participating in local immune responses. Moreover, the spleen may modulate the systemic immune status skin neuroendocrine system, which can also be known as the “Neuro-Immune-Skin Axis” from the research on psoriasis and atopic dermatitis (Song et al. 2021; Shirley et al. 2024). We hypothesized that the CXCR6 + γδT cells and CXCR6 + NKT cells might express inflammatory factors and chemokines, resulting in direct or indirect injury to melanocytes. Damaged melanocytes secrete exosomes that contain melanocyte-specific antigens and damage-associated molecular patterns (DAMPs), which may induce the maturation of nearby dendritic cells into efficient antigen-presenting cells (APCs). These APCs may migrate to peripheral lymph nodes and the spleen, leading to activation of specific immunity. Our results also indicate that the CXCL16-CXCR6 axis plays a specific role in the initiation of chronic mental stress-induced vitiligo, linking stromal cells with immune cells, resident immune cells (skin), and a distant immune organ (spleen).

Although there is a strong link between vitiligo and immunity, patients with vitiligo usually do not have dense lymphocyte infiltration, such as that seen in melanoma or in response to treatments that block PD-1. Our previous ultra-microscopy observations (Ding et al. 2015) revealed mitochondria damage in melanocytes and keratinocytes of vitiligo with halo naevus that was absent in vitiligo without halo naevus. These observations indicate that active immunity-related vitiligo might have disparate mechanisms compared with vitiligo without active immunity events, at least in the early stage.

Despite some evidence of local CD8 + cytotoxic T lymphocyte infiltration in vitiligo, pathological biopsies usually show sparse or sometimes absent local lymphocyte infiltration (Picardo et al. 2015), which is markedly different from the pattern observed in common clinical autoimmune diseases, for example, cutaneous lupus erythematosus (Niebel et al. 2023), where lymphatic infiltration is typically present in lesions. Consistent with vitiligo, we observed little lymphatic infiltration in the tail skin leukoderma of CUMS mice. Moreover, the number of inflammatory cells in vitiligo is even lower than that in psoriasis (Griffiths et al. 2021), another Th17 cell-mediated innate immune disease, indicating that vitiligo exhibits a unique immune phenomenon.

Immunosuppressive medications such as hormones are effective (Mukhatayev and Le Poole 2024), while alternatives like Vitamin D3 derivatives and topical applications of traditional Chinese medicines can protect melanocytes and promote repigmentation, probably via non-immune pathways (Gong et al. 2015). Studies have revealed unbiased with the elevated levels of cytokines, such as Th1, Th2, and Th17, in the serum of vitiligo patients (Gholijani et al. 2020; Kądziela et al. 2024). Therefore, we suggest that vitiligo cannot be simply classified as a specific type of autoimmune disease, but is a heterogeneous immune disorder. Moreover, no monoclonal antibody has been developed that can effectively treat most vitiligo patients (Kądziela et al. 2024).

The pathogenesis and progression of vitiligo with a strong immune response, such as in the presence of halo naevus or melanoma, may be different from that of vitiligo without these comorbidities. Most mouse models used in basic vitiligo research simulate post-melanoma leukoderma; therefore, it is crucial to develop a vitiligo model that is closer to the human state and that does not have an obvious trigger of CD8 + cytotoxic T lymphocyte cytotoxicity for melanocyte-specific killing.

Our study has some limitations. First, the seq-RNA sample size is relatively small (three versus three) and they are derived from a single time point, providing only a snapshot rather than following the hair growth cycle. Second, immunohistochemistry was the sole method used for verification, and we did not generate functional assays in vivo or flow cytometry in vitro evidence. This could be approached by using the Cre-loxP system for gene editing to enable live-tracing observations, or the cultivation of organoids to model the internal environment.

## Conclusions

The occurrence and progression of vitiligo are influenced not only by cytotoxic T lymphocyte infiltration but also by other factors, such as melanocyte detachment through the skin, melanocyte senescence, melanocyte pyroptosis, and ferroptosis (Qiu et al. 2017; Jin et al. 2021). Our CUMS-leukoderma mouse model has a similar inflammatory and immune microenvironment to human vitiligo and is valuable for the comprehensive analysis of vitiligo induced by chronic mental stress. Our findings describe the microenvironment and fate of two clusters of melanocytes (Mel2 and Mel3). Additionally, CXCL16-CXCR6 is found to be the main ligand-receptor pair that links innate immunity and adaptive immunity, which might be the vital molecule in the initiation of the antigen-presenting capacity of APC. The non-specific immune-killing mediated by γδ T cells and NKT cells, rather than by cytotoxic T lymphocytes in the early stage, and subsequent melanocyte-associated-antigen exposure to APCs is supposed to trigger adaptive immune response. Hypothesis schematics of our CUMS model is shown in Fig. 10. Nevertheless, further research is needed to elucidate the underlying molecular mechanism of this process.

## Supplementary Information

Supplementary Material 1: Fig S1. Quality control of our scRNASeq analysis protocol. A. Dimplot of the identified cells. B. Scatterplot of the correlation analysis between the number of UMI and the number of genes. The dots of different colors represent cells from different samples. The X-axis is the number of UMIs, the Y-axis is the number of genes, and the number above the figure is the Pearson correlation coefficient. C. Violin plot of the distribution of basic cell information in each sample after filtration. Left is the distribution of the number of genes detected in a single cell of each sample; middle is the distribution of the total number of UMI detected in a single cell of each sample; right is the percentage distribution of mitochondrial gene expression in individual cells of each sample. Abbreviations: IB, inner bulge; KC, keratinocytes; OB, outer bulge; SG, Sebaceous gland; UFC, upper hair follicle cells

Supplementary Material 2: FigS2. Characterization of the Mel2 and Mel3 clusters in mouse skin by scRNASeq. A. UMAP for four melanocyte sub-clusters, Mel1–Mel4. B. Dot plot displaying top expressed genes in each cell population. C. Heatmap of Top 5 marker gene expression. D. Enrichment analysis of Mel2 cells. E. Enrichment analysis of Mel3 cells

Supplementary Material 3: FigS3. Characterization of the hair follicle cells in mouse skin by scRNASeq. A. UMAP for Hair follicle cells. B. Dot plot displaying top expressed genes in each cell population. C. Box plot the distribution of four selected cell populations D. Heatmap of Top 5 marker gene expression. E. HFSC associated cytokines. F. Genes related to the γ-interferon pathway in each group. G. Genes related to the γ-interferon pathway in different cell cluster. Each sample is represented as one dot. P-values were calculated using two-sided Mann–Whitney U-test. *p < 0.05. Abbreviations: HFSC, hair follicle stem cell; IB, inner bulge; KC, keratinocytes; OB, outer bulge; SG, Sebaceous gland; UFC, upper hair follicle cells

Supplementay Material 4: FigS4. Characterization of the Keratinocyte and Fibroblast in mouse tail skin by scRNASeq. A. UMAP for Keratinocytes. B. Dot plot displaying top expressed genes in each cell population. C. Box plot the distribution of four selected cell populations D. GSVA for cell death modes and related pathways in each group. E. UMAP for fibroblasts. F. Dot plot displaying top expressed genes in each cell population. G. Box plot the distribution of two selected cell populations. H. Fibroblasts associated cytokines. Each sample is represented as one dot. P-values were calculated using two-sided Mann–Whitney U-test. *p < 0.05. Abbreviations: FC, fibroblasts; KC, keratinocytes

Supplementary Material 5: FigS5. Cellchat of the different cells with the Mel2 and Mel3 clusters from the mouse skin. A. Overall comparisons of interaction number and strength between control group and CUMS group. B. Dot plot showed the comparison of the multiple ligand–receptor pairs between Mel2 cluster and other cells. C. Dot plot showed the comparison of the multiple ligand–receptor pairs between Mel3 cluster and other cells. Abbreviations: HFSC, hair follicle stem cell; IB, inner bulge; KC, keratinocytes; OB, outer bulge; SG, Sebaceous gland; UFC, upper hair follicle cells

Supplementary Material 6: FigS6. Immunofluorescence of immune cells from mouse spleen. A. NKT cells marked with NKR-P1 C and immune cells marked with increased in CUMS, CXCR3 also increased in CUMS. DAPI, CD3, NKR-P1 C, CXCR6. B.CD8 + Tem marked with CD8, CD103 and CD127 increased in CUMS. DAPI, CD103, CD8, CD127. scale bar = 50 μm. Data are from three independent experiments. Abbreviations: NKT, natural killer T cells; Tem, effector memory T cells

Supplementary Material 7

## Data Availability

The data that support the findings of this study are available from the corresponding author upon reasonable request.

## Abbreviations

APM: Arrector pili muscle
CUMS: Chronic unpredictable mild stress
DAMPs: Damage-associated molecular patterns
DC: Dendritic cells
FC: Fibroblasts
FST: Forced Swimming Test
HFSC: Hair follicle stem cell
IB: Inner bulge
KC: Keratinocytes
Mac: Macrophages
MC: Melanocyte
McSC: Melanocyte stem cell
M1: Macrophages M1 pro-inflammatory phenotype
NKT: Natural killer T cells
TST: Tail Suspension Test
SPT: Sucrose Preference Test
OB: Outer bulge
OFT: Open Field Test
SG: Sebaceous gland
Tcyt: Cytotoxic T cells
Tem: Effector memory T cells
Th1: Helper T cells
Treg: Regulatory T cells
Trm: Resident memory T cells
UFC: Upper hair follicle cells (infundibulum)

## References

[CR1] Aaron.B. Lerner, Shiohara T, Boissy R.E, Jacobson K.A, Lamoreux M.L, Moellmann G.E. A mouse model for vitiligo, J. Invest. Dermatol. 1986;87:299–304. 10.1111/1523-1747.ep12524353 3525691

[CR2] Angel P, Szabowski A, Schorpp-Kistner M. Function and regulation of AP-1 subunits in skin physiology and pathology. Oncogene. 2001;30(20):2413–23. 10.1038/sj.onc.1204380 11402337

[CR3] Bibeau K, Ezzedine K, Harris JE, van Geel N, Grimes P, Parsad D, et al. Mental Health and Psychosocial Quality-of-Life Burden Among Patients with Vitiligo: Findings from the Global VALIANT Study. JAMA Dermatol. 2023;159:1124–8. 37647073 10.1001/jamadermatol.2023.2787 PMC10469285

[CR4] Bisgaard TH, Allin KH, Keefer L, Ananthakrishnan AN, Jess T. Depression and anxiety in inflammatory bowel disease: epidemiology, mechanisms and treatment. Nat Rev Gastroenterol Hepatol. 2022;19:717–26. 35732730 10.1038/s41575-022-00634-6

[CR5] Cai X, Han M, Lou F, Sun Y, Yin Q, Sun L, et al. Tenascin C(+) papillary fibroblasts facilitate neuro-immune interaction in a mouse model of psoriasis. Nat Commun. 2023;14:2004. 37037861 10.1038/s41467-023-37798-x PMC10086024

[CR6] Ding GZ, Zhao WE, Li X, Gong QL, Lu Y. A comparative study of mitochondrial ultrastructure in melanocytes from perilesional vitiligo skin and perilesional halo nevi skin. Arch Dermatol Res. 2015;307:281–9. 25672813 10.1007/s00403-015-1544-4

[CR7] Eby JM, Kang HK, Klarquist J, Chatterjee S, Mosenson JA, Nishimura MI, et al. Immune responses in a mouse model of vitiligo with spontaneous epidermal de- and repigmentation. Pigm Cell Melanoma r. 2014;27:1075–85. 10.1111/pcmr.12284 PMC4470702 24935676

[CR8] Erdoğan A, Mutlu HS, Solakoğlu S. Autologously transplanted dermis-derived cells alleviated monobenzone-induced vitiligo in mouse. Exp Dermatol. 2022;31:1355–63. 35538739 10.1111/exd.14603

[CR9] Ezzedine K, Eleftheriadou V, Whitton M, van Geel N. Vitiligo Lancet. 2015;386:74–84. 25596811 10.1016/S0140-6736(14)60763-7

[CR10] Frisoli ML, Essien K, Harris JE. Vitiligo: Mechanisms of Pathogenesis and Treatment. ANNU Rev Immunol. 2020;38:621–48. 32017656 10.1146/annurev-immunol-100919-023531

[CR11] Gholijani N, Yazdani MR, Dastgheib L. Predominant role of innate pro-inflammatory cytokines in vitiligo disease. Arch Dermatol Res. 2020;312:123–31. 31620869 10.1007/s00403-019-01996-9

[CR12] Gong Q, Li X, Sun J, Ding G, Zhou M, Zhao W, et al. The effects of calcipotriol on the dendritic morphology of human melanocytes under oxidative stress and a possible mechanism: is it a mitochondrial protector? J Dermatol Sci. 2015;77:117–24. 25592908 10.1016/j.jdermsci.2014.12.006

[CR13] Gregg RK, Nichols L, Chen Y, Lu B, Engelhard VH. Mechanisms of Spatial and Temporal Development of Autoimmune Vitiligo in Tyrosinase-Specific TCR Transgenic Mice. J Immunol. 2010;184:1909–17. 20083666 10.4049/jimmunol.0902778 PMC2887735

[CR14] Griffiths C, Armstrong AW, Gudjonsson JE, Barker J. Psoriasis Lancet. 2021;397:1301–15. 33812489 10.1016/S0140-6736(20)32549-6

[CR15] He H, Suryawanshi H, Morozov P, Gay-Mimbrera J, Del DE, Kim HJ, et al. Single-cell transcriptome analysis of human skin identifies novel fibroblast subpopulation and enrichment of immune subsets in atopic dermatitis. J Allergy Clin Immunol. 2020;145:1615–28. 32035984 10.1016/j.jaci.2020.01.042

[CR16] Huang L, Zuo Y, Li S, Li C. Melanocyte stem cells in the skin: Origin, biological characteristics, homeostatic maintenance and therapeutic potential. Clin Transl Med. 2024;14:e1720. 38778457 10.1002/ctm2.1720 PMC11111606

[CR17] Jin S, Guerrero-Juarez CF, Zhang L, Chang I, Ramos R, Kuan CH, et al. Inference and analysis of cell-cell communication using Cell Chat. Nat Commun. 2021;12:1088. 33597522 10.1038/s41467-021-21246-9 PMC7889871

[CR18] Johnson R, Jackson IJ. Light is a dominant mouse mutation resulting in premature cell death. Nat Genet. 1992;1:226–9. 1303241 10.1038/ng0692-226

[CR19] Kądziela M, Kutwin M, Karp P, Woźniacka. A Role of Cytokines and Chemokines in Vitiligo and Their Therapeutic Implications. J Clin Med. 2024;13:4919 10.3390/jcm13164919 PMC11355229 39201060

[CR20] Kang P, Wang Y, Chen J, Chang Y, Zhang W, Cui T, et al. TRPM2-dependent autophagy inhibition exacerbates oxidative stress-induced CXCL16 secretion by keratinocytes in vitiligo. J Pathol. 2024;262:441–53. 38186269 10.1002/path.6247

[CR21] Lai YC, Yew YW, Kennedy C, Schwartz RA. Vitiligo and depression: a systematic review and meta- analysis of observational studies. Br J Dermatol. 2017;177:708–18. 27878819 10.1111/bjd.15199

[CR22] Li S, Zhu G, Yang Y, Jian Z, Guo S, Dai W, et al. Oxidative stress drives CD8(+) T-cell skin trafficking in patients with vitiligo through CXCL16 upregulation by activating the unfolded protein response in keratinocytes. J Allergy Clin Immunol. 2017;140:177–89. 27826097 10.1016/j.jaci.2016.10.013

[CR23] Markov DD, Novosadova EV. Chronic Unpredictable Mild Stress Model of Depression: Possible Sources of Poor Reproducibility and Latent Variables. Biology (Basel). 2022;11:1621. 36358321 10.3390/biology11111621 PMC9687170

[CR24] Mehrotra S, Al-Khami AA, Klarquist J, Husain S, Naga O, Eby JM, et al. A Coreceptor-Independent Transgenic Human TCR Mediates Anti-Tumor and Anti-Self Immunity in Mice. J Immunol. 2012;189:1627–38. 22798675 10.4049/jimmunol.1103271 PMC3674773

[CR25] Mukhatayev Z, Le Poole IC. Vitiligo: advances in pathophysiology research and treatment development. Trends Mol Med. 2024;30:844–62. 38705825 10.1016/j.molmed.2024.04.009

[CR26] Niebel D, de Vos L, Fetter T, Brägelmann C, Wenzel J. Cutaneous Lupus Erythematosus: An Update on Pathogenesis and Future Therapeutic Directions. Am J Clin Dermatol. 2023;24:521–40. 37140884 10.1007/s40257-023-00774-8 PMC10157137

[CR27] Picardo M, Dell’Anna ML, Ezzedine K, Hamzavi I, Harris JE, Parsad D, et al. Vitiligo Nat Rev Dis Primers. 2015;1:15011. 27189851 10.1038/nrdp.2015.11

[CR28] Plikus MV, Wang X, Sinha S, Forte E, Thompson SM, Herzog EL, et al. Fibroblasts: Origins, definitions, and functions in health and disease. Cell. 2021;184:3852–72. 34297930 10.1016/j.cell.2021.06.024 PMC8566693

[CR29] Qiu X, Mao Q, Tang Y, Wang L, Chawla R, Pliner HA, et al. Reversed graph embedding resolves complex single-cell trajectories. Nat Methods. 2017;14:979–82. 28825705 10.1038/nmeth.4402 PMC5764547

[CR30] Shi Z, Liu Z, Wei Y, Zhang R, Deng Y, Li D. The role of dermal fibroblasts in autoimmune skin diseases. FRONT IMMUNOL. 2024;15:1379490. 38545113 10.3389/fimmu.2024.1379490 PMC10965632

[CR31] Shirley SN, Watson AE, Yusuf N. Pathogenesis of Inflammation in Skin Disease: From Molecular Mechanisms to Pathology. Int J Mol Sci. 2024;25(18):10152. 10.3390/ijms251810152 PMC11431851 39337637

[CR32] Song Z, Zhang J, Yu JJ, Chen XL, Zhang FY, Wei W, et al. Hyperforin Ameliorates Imiquimod-Induced Psoriasis-Like Murine Skin Inflammation by Modulating IL-17A-Producing γδ T Cells. Front Immunol. 2021;5(12):635076. 10.3389/fimmu.2021.635076 PMC8131513 34025642

[CR33] Tulic MK, Cavazza E, Cheli Y, Jacquel A, Luci C, Cardot-Leccia N, et al. Innate lymphocyte-induced CXCR3B-mediated melanocyte apoptosis is a potential initiator of T-cell autoreactivity in vitiligo. Nat Commun. 2019;10:2178. 31097717 10.1038/s41467-019-09963-8 PMC6522502

[CR34] Urtatiz O, Haage A, Tanentzapf G, Van Raamsdonk CD. Crosstalk with keratinocytes causes GNAQ oncogene specificity in melanoma. Life. 2021;10:e71825. 10.7554/eLife.71825 PMC8747508 34939927

[CR35] Vallerand IA, Lewinson RT, Parsons LM, Hardin J, Haber RM, Lowerison MW, et al. Vitiligo and major depressive disorder: A bidirectional population-based cohort study. J Am Acad Dermatol. 2019;80:1371–9. 30528503 10.1016/j.jaad.2018.11.047

[CR36] Wu X, Yang Y, Xiang L, Zhang C. The fate of melanocyte: Mechanisms of cell death in vitiligo Pigment. Cell Melanoma Res. 2021;34:256–67. 10.1111/pcmr.12955 33346939

[CR37] Wu W, Wang X, He K, Li C, Li S. From mice to men: An assessment of preclinical model systems for the study of vitiligo. CLIN IMMUNOL. 2024;262:110171. 38462156 10.1016/j.clim.2024.110171

[CR38] Xu Z, Chen D, Hu Y, Jiang K, Huang H, Du Y, et al. Anatomically distinct fibroblast subsets determine skin autoimmune patterns. Nature. 2022;601:118–24. 34912121 10.1038/s41586-021-04221-8

[CR39] Yang P, Luan M, Li W, Niu M, He Q, Zhao Y, et al. Single-cell transcriptomics reveals peripheral immune responses in non-segmental vitiligo. Front Immunol. 2023;14:1221260. 38077333 10.3389/fimmu.2023.1221260 PMC10702986

[CR40] Zhang B, Chen T. Local and systemic mechanisms that control the hair follicle stem cell niche. Nat Rev Mol Cell Biol. 2024;25:87–100. 37903969 10.1038/s41580-023-00662-3

[CR41] Zhang L, Yu X, Zheng L, Zhang Y, Li Y, Fang Q, et al. Lineage tracking reveals dynamic relationships of T cells in colorectal cancer. Nature. 2018;564:268–72. 30479382 10.1038/s41586-018-0694-x

[CR42] Zhang B, Ma S, Rachmin I, He M, Baral P, Choi S, et al. Hyperactivation of sympathetic nerves drives depletion of melanocyte stem cells. Nature. 2020;577:676–81. 31969699 10.1038/s41586-020-1935-3 PMC7184936

[CR43] Zang D, Niu C, Lu X, Aisa HA. A Novel Furocoumarin Derivative, 5-((diethylamino)me-13 thyl)-3-phenyl-7H-furo [3,2-g] chromen-7-one Upregulates Melanin Synthesis via the Activation of cAMP/PKA and MAPKs Signal Pathway: In Vitro and In Vivo Study. Int J Mol Sci. 2022;23:14190. 10.3390/ijms232214190 PMC9694462 36430668

[CR44] Zhang H, Wang M, Zhao X, Wang Y, Chen X, Su J. Role of stress in skin diseases: A neuroendocrine-immune interaction view. Brain Behav Immun. 2024;116:286–302. 38128623 10.1016/j.bbi.2023.12.005

[CR45] Zhang J, Li J, Huang J, Xiang X, Li R, Zhai Y, et al. Psychological stress disturbs bone metabolism via miR-335–3p/Fos signaling in osteoclast. Elife. 2025;8(13):RP95944. 10.7554/eLife.95944 PMC11709429 39773351

[CR46] Zhou L, Li K, Shi YL, Hamzavi I, Gao TW, Henderson M, et al. Systemic analyses of immunophenotypes of peripheral T cells in non-segmental vitiligo: implication of defective natural killer T cells. Pigment Cell Melanoma Res. 2012;25:602–11. 22591262 10.1111/j.1755-148X.2012.01019.x PMC3801166

