# Supplementary figures and images for: Comprehensive single-cell transcriptomic reveals different destinies of melanocytes and dynamic changes of immune microenvironment in a psychological stress-induced leukoderma and leukotrichia mouse model
Source: Mol Med. 2025 May 14;31:186. doi: 10.1186/s10020-025-01236-z (PMC12076869; doi:10.1186/s10020-025-01236-z)

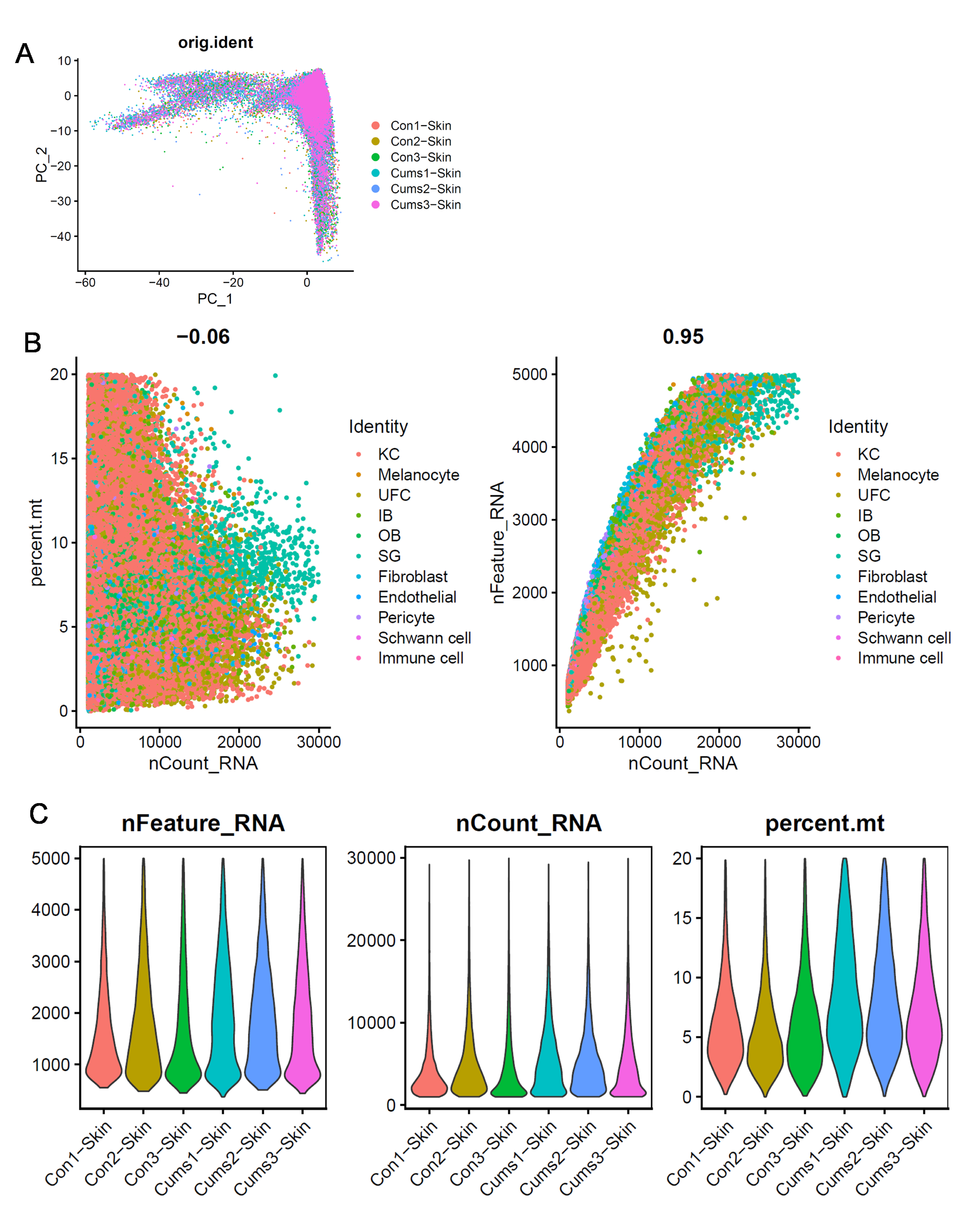

Supplement: Supplementary file 1 — Supplementary Material 1: Fig S1. Quality control of our scRNASeq analysis protocol. A. Dimplot of the identified cells. B. Scatterplot of the correlation analysis between the number of UMI and the number of genes. The dots of different colors represent cells from different samples. The X-axis is the number of UMIs, the Y-axis is the number of genes, and the number above the figure is the Pearson correlation coefficient. C. Violin plot of the distribution of basic cell information in each sample after filtration. Left is the distribution of the number of genes detected in a single cell of each sample; middle is the distribution of the total number of UMI detected in a single cell of each sample; right is the percentage distribution of mitochondrial gene expression in individual cells of each sample. Abbreviations: IB, inner bulge; KC, keratinocytes; OB, outer bulge; SG, Sebaceous gland; UFC, upper hair follicle cells [file 10020_2025_1236_MOESM1_ESM.png]

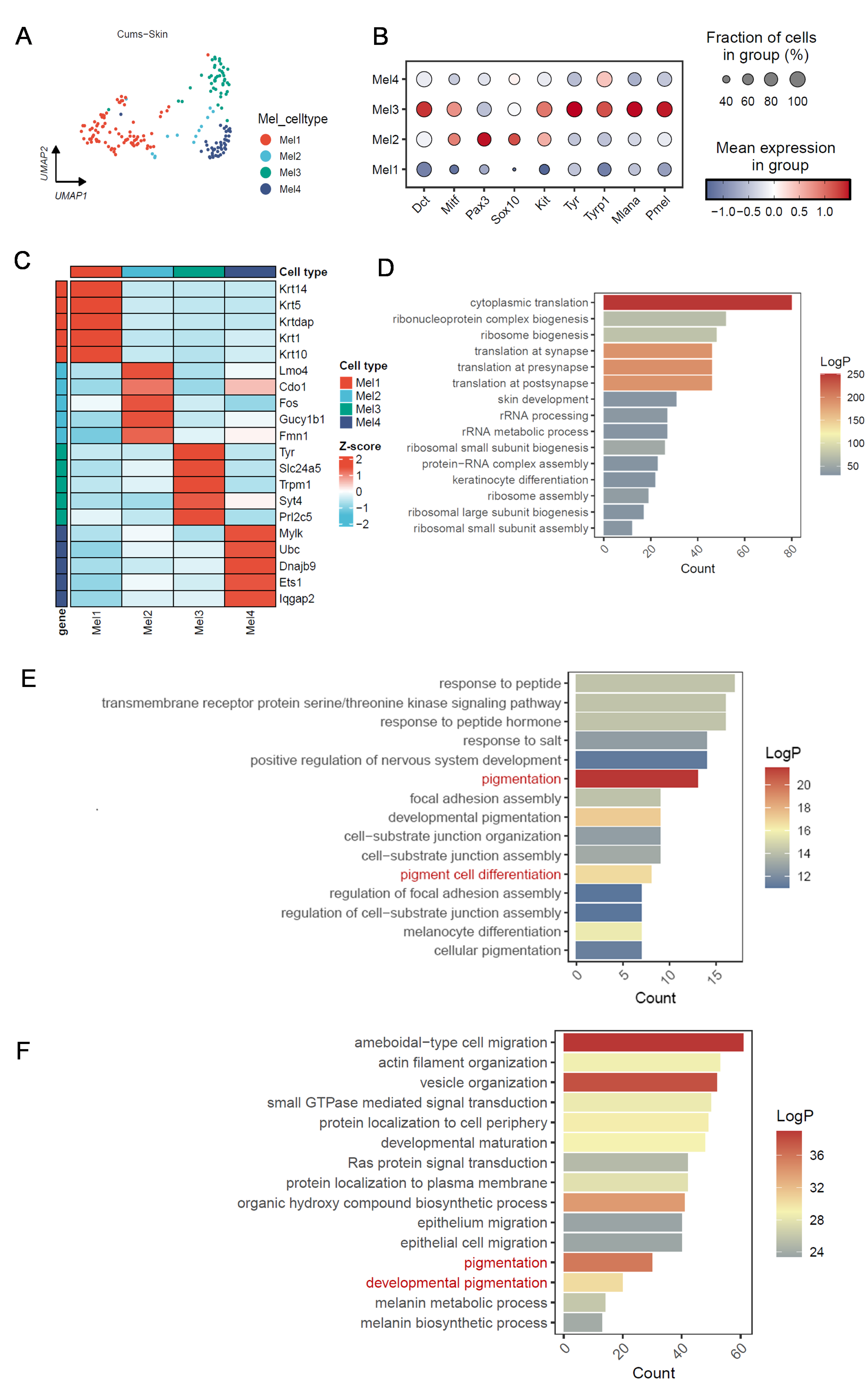

Supplement: Supplementary file 2 — Supplementary Material 2: FigS2. Characterization of the Mel2 and Mel3 clusters in mouse skin by scRNASeq. A. UMAP for four melanocyte sub-clusters, Mel1–Mel4. B. Dot plot displaying top expressed genes in each cell population. C. Heatmap of Top 5 marker gene expression. D. Enrichment analysis of Mel2 cells. E. Enrichment analysis of Mel3 cells [file 10020_2025_1236_MOESM2_ESM.png]

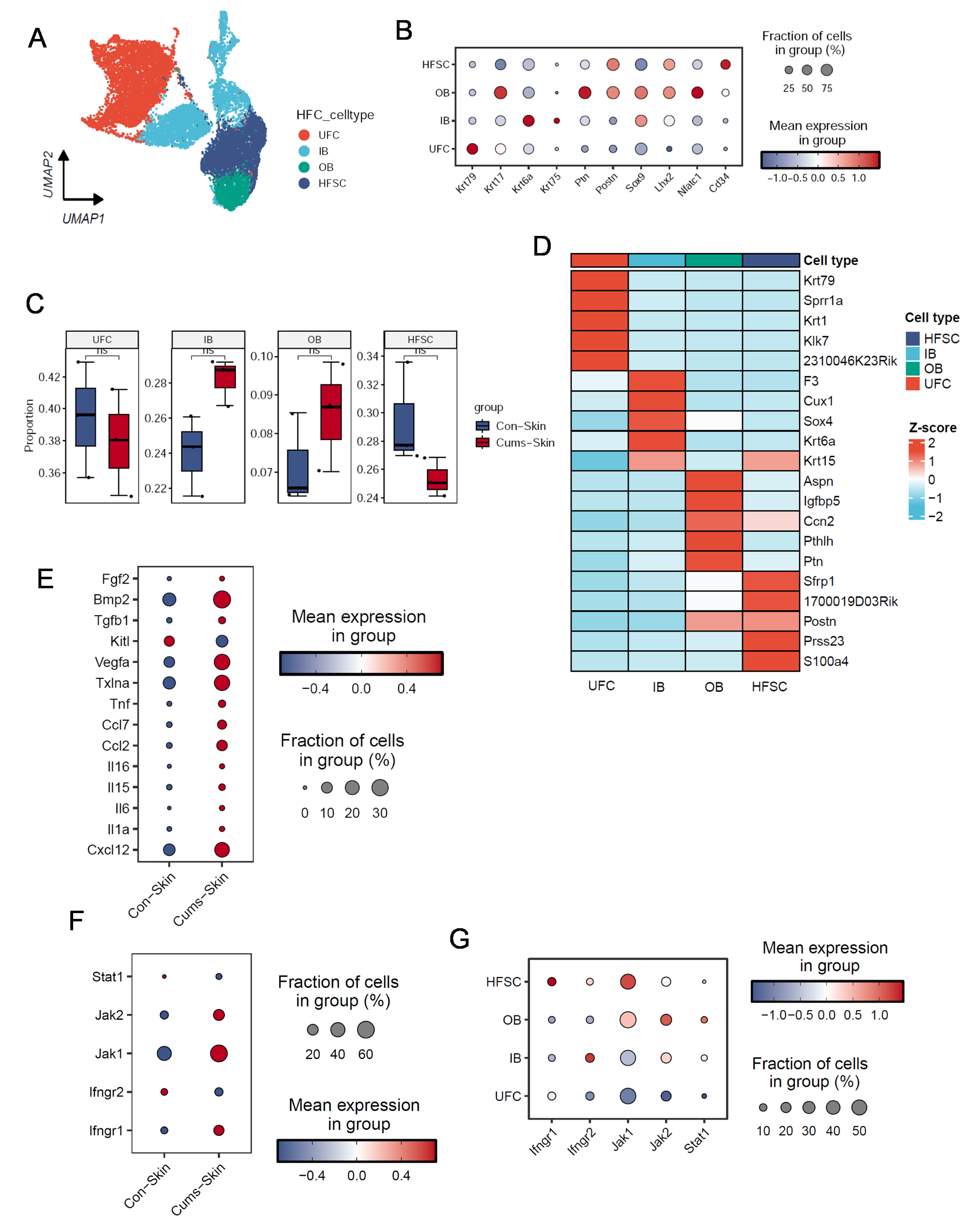

Supplement: Supplementary file 3 — Supplementary Material 3: FigS3. Characterization of the hair follicle cells in mouse skin by scRNASeq. A. UMAP for Hair follicle cells. B. Dot plot displaying top expressed genes in each cell population. C. Box plot the distribution of four selected cell populations D. Heatmap of Top 5 marker gene expression. E. HFSC associated cytokines. F. Genes related to the γ-interferon pathway in each group. G. Genes related to the γ-interferon pathway in different cell cluster. Each sample is represented as one dot. P-values were calculated using two-sided Mann–Whitney U-test. *p < 0.05. Abbreviations: HFSC, hair follicle stem cell; IB, inner bulge; KC, keratinocytes; OB, outer bulge; SG, Sebaceous gland; UFC, upper hair follicle cells [file 10020_2025_1236_MOESM3_ESM.png]

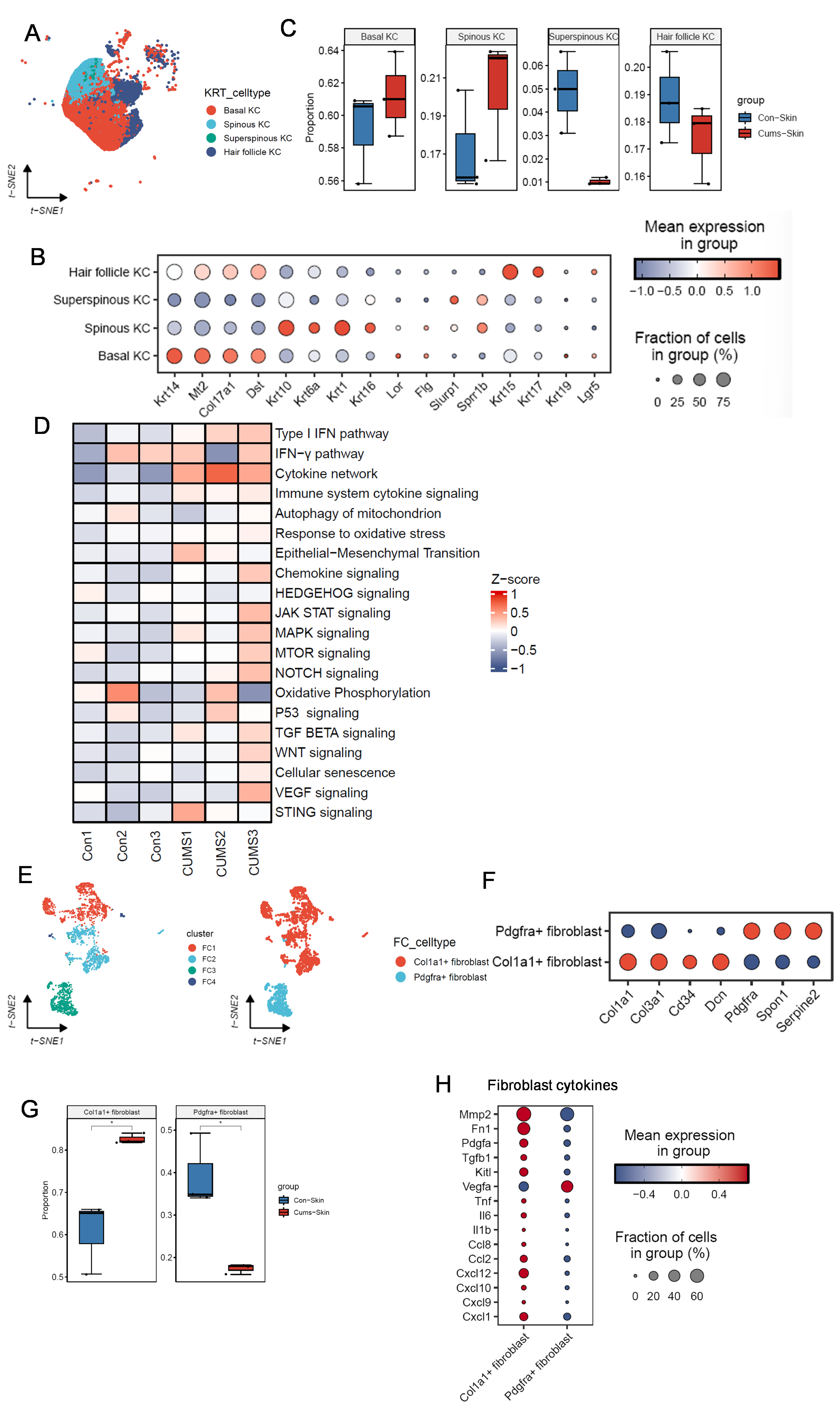

Supplement: Supplementary file 4 — Supplementay Material 4: FigS4. Characterization of the Keratinocyte and Fibroblast in mouse tail skin by scRNASeq. A. UMAP for Keratinocytes. B. Dot plot displaying top expressed genes in each cell population. C. Box plot the distribution of four selected cell populations D. GSVA for cell death modes and related pathways in each group. E. UMAP for fibroblasts. F. Dot plot displaying top expressed genes in each cell population. G. Box plot the distribution of two selected cell populations. H. Fibroblasts associated cytokines. Each sample is represented as one dot. P-values were calculated using two-sided Mann–Whitney U-test. *p < 0.05. Abbreviations: FC, fibroblasts; KC, keratinocytes [file 10020_2025_1236_MOESM4_ESM.png]

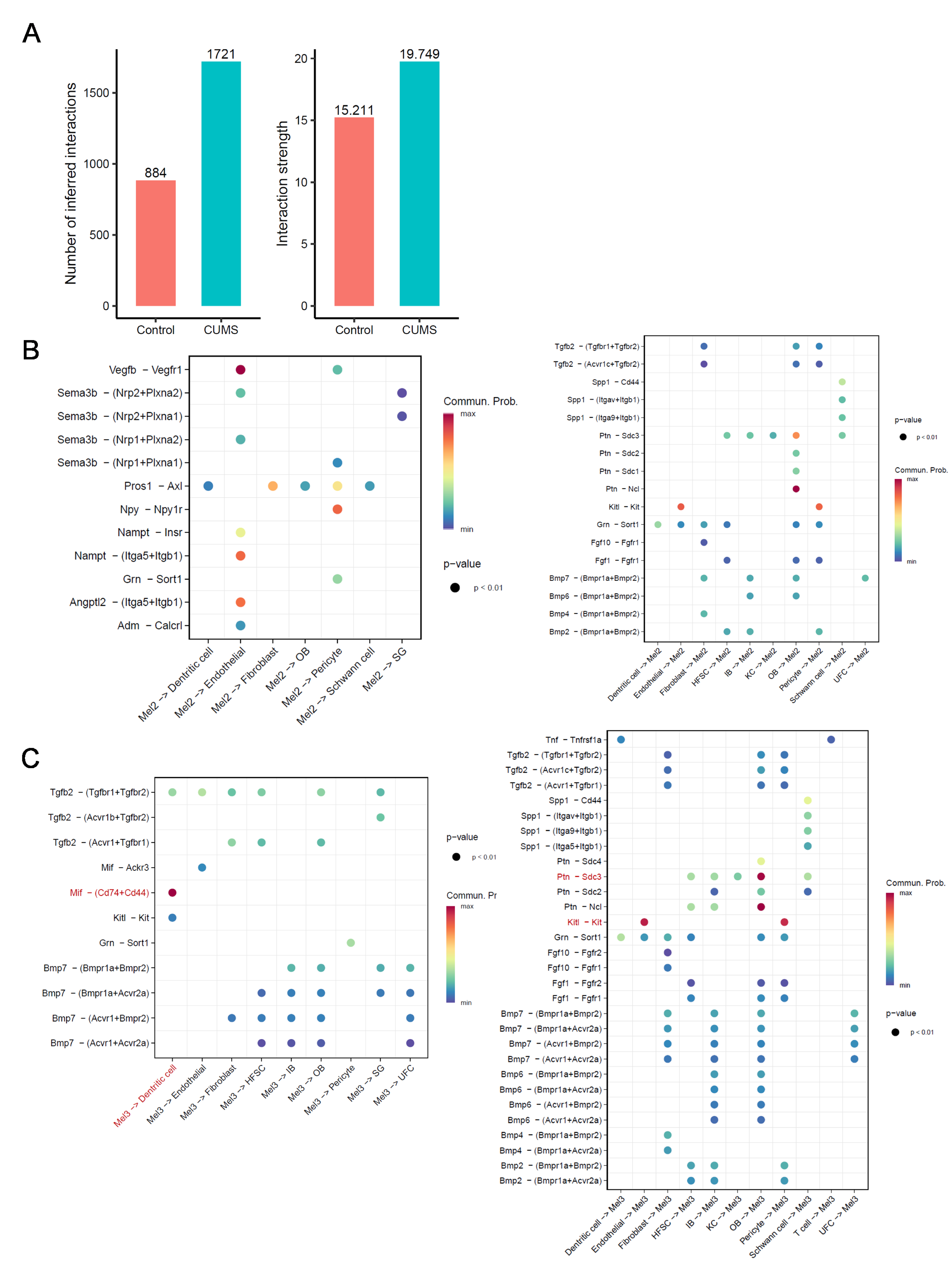

Supplement: Supplementary file 5 — Supplementary Material 5: FigS5. Cellchat of the different cells with the Mel2 and Mel3 clusters from the mouse skin. A. Overall comparisons of interaction number and strength between control group and CUMS group. B. Dot plot showed the comparison of the multiple ligand–receptor pairs between Mel2 cluster and other cells. C. Dot plot showed the comparison of the multiple ligand–receptor pairs between Mel3 cluster and other cells. Abbreviations: HFSC, hair follicle stem cell; IB, inner bulge; KC, keratinocytes; OB, outer bulge; SG, Sebaceous gland; UFC, upper hair follicle cells [file 10020_2025_1236_MOESM5_ESM.png]
